# 

**Published:** 1797

**Authors:** 


LONDON:

PRINTED FOR J. JOHNSON,
NO. 72, ST. PAUL'S CHURCH YARD.

M.DCC.XCVII.

1797

*Index.* — — --- 372

---

DIRECTIONS TO THE BINDER.

Plate the Firſt may be placed at page 92, Plate the Second, at page 112; and Plate the Third, at page 217.

### CATALOGUE OF BOOKS.

1. A TREATISE upon Gravel and upon Gout, in which their Sources and Connection are ascertained; with an Examination of Dr. Austin's Theory of Stone, and other Critical Remarks. A Dissertation on the Bile, and its Concretions; and an Inquiry into the Operation of Solvents. By *Murray Forbes*, Member of the Surgeon's Company. 8vo. *Cadell*, London, 1793.

2. A Case of Hydrophobia, commonly called Canine Madness, from the Bite of a Mad Dog, successfully treated. By *Thomas Arnold*, M. D. Fellow of the Royal College of Physicians and of the Royal Medical Society of Edinburgh. 8vo. *Dilly*, London, 1793.

3. The Art of preventing Diseases, and restoring Health, founded on Rational Principles, and adapted to Persons of every Capacity. By *George Wallis*, M. D. 8vo. *Robinsons*, London, 1793.

4. Advice to Parents on the Management of Children in the Natural Small Pox. 8vo. *Robinsons*, London, 1793.

5. A Trea-

5. A Treatife on the Errors and Defects of Medical Education; in which are contained Obfervations on the Means of correcting them. By *Thomas Withers*, M. D. M. M. S. L. Phyfician to the York County Hofpital. 8vo. *Dilly*, London, 1794.

6. An Effay on the Management, Nurfing, and Difeafes of Children, from the Birth; and on the Treatment and Difeafes of pregnant and lying-in Women: with Remarks on the domeftic Practice of Medicine. The fecond Edition revifed and confiderably enlarged. To which is now added, the Treatment and Difeafes of Children at more advanced Periods of Childhood; with Obfervations on Mothers nurfing their Children. By *William Mofs*, Surgeon to the Liverpool Lying-in Charity. 8vo. *Longman*, London, 1794.

7. An Account of a new and fuccefsful Method of treating thofe Affections which arife from the Poifon of Lead; to which are added general Obfervations on the internal Ufe of Lead as a Medicine. By *Henry Clutterbuck*, Member of the Corporation of Surgeons, &c. 8vo. *Boofey*, London, 1794.

8. Letters, concerning the internal Dropfy of the Brain, to Charles William Quin, M. D.

Fellow

Fellow of the King's and Queen's College of Phyficians, Phyfician General of His Majefty's Army in Ireland, and of the Royal Hofpital for Invalids, near Dublin, from *William Patterfon*, M. D. Member of the Royal Irifh Academy, and Correfponding Member of the London Medical Society. 8vo. Dublin, 1794.

9. Remarks on fome of the Opinions of Dr. Rufh, refpecting the Yellow Fever which prevailed in Philadelphia in 1793. By *William Patterfon*, M. D. 8vo. Londonderry, 1795.

10. A Differtation on Simple Fever, or on Fevers confifting of one Paroxyfm only. By *George Fordyce*, M. D. F. R. S. Senior Phyfician to St. Thomas's Hofpital. 8vo. *Johnfon*, London, 1794.

11. A Second Differtation on Fever, containing the Hiftory and Method of Treatment of a regular Tertian Intermittent. By *George Fordyce*, M. D. F. R. S. &c. 8vo. *Johnfon*, London, 1795.

12. An Addrefs to Medical Students; a Letter to Dr. Fordyce; with Remarks and Queftions upon Quotations from Dr. Fordyce's Differtation on Simple Fever. 8vo. *Bell*, London, 1795.

13. The Phyfician's Vade Mecum; being a Compendium of Nofology and Therapeutics, for for the Use of Students. By the Rev. *Joseph Townsend*, Rector of Pewsey, Wilts; and Author of a Tour through Spain. 12mo. *Dilly*, London, 1794.

14. A Guide to Health; being Cautions and Directions in the Treatment of Diseases. Designed chiefly for the Use of Students. By the Rev. *Joseph Townsend*, Rector of Pewsey, Author of the Physician's Vade Mecum, and of a Journey through Spain. 8vo. *Cox*, London, 1795.

15. Catechism of Health: for the Use of Schools, and for Domestic Instruction. By *B. C. Faust*, M. D. Counsellor and Physician to the Reigning Count of Schaumburgh Lippe, &c. translated from the last improved German Edition of this Work by *J. H. Basse*. 12mo. *Dilly*, London, 1794.

16. The rational and improved Practice of Physic. By *William Rowley*, M. D. Member of the University of Oxford, the Royal College of Physicians in London, and Physician to the St. Mary-le-Bone Infirmary. 4 vols. 8vo. *Newbery*, London, 1794.

17. Medical Histories and Reflections. Volume* the Second. By *John Ferriar*, M. D. Physician to the Manchester Infirmary, Dispen-

* See Vol. IV. p. 205.

fary, Lunatic Hofpital, and Afylum. 8vo. *Cadell* and *Davies*, London, 1795.

18. A Treatife on the Nature and Cure of the Cynanche Trachealis, commonly called the Croup. By *Difney Alexander*, Member of the Royal Medical Society of Edinburgh. 8vo. *Johnfon*, London, 1794.

19. Obfervations on the Tuffis convulfiva, or Hooping Cough; as read at the Lyceum Medicum Londinenfe: wherein the Nature, Caufe, and Cure of this Difeafe are endeavoured to be demonftrated, and the Practice of giving Emetics fhewn to be pernicious and ufelefs. By *John Gale Jones*. 8vo. *Allen* and *Weft*, London, 1795.

20. Obfervations, anatomical, phyfiological, and pathological, on the pulmonary Syftem: with Remarks on fome of the Difeafes of the Lungs, viz. Haemorrhage, Wounds, Afthma, Catarrh, Croup, and Confumption; tending to eftablifh a New Pathology of the Lungs, founded on the Anatomy and Phyfiology of the Parts. Some Remarks are introduced on the Broken-wind of Horfes. And to the whole is added an Appendix, containing Obfervations on fome of the Articles of the Materia Medica, viz. on the Rofa Rubra, Flores Chamæmeli and Sarfaparilla; as alfo on the Cicuta, Stramonium, Hyofciamus, and Aconitum.

nitum. By *William Davidson.* 8vo. *Egerton*, London, 1795.

21. The Elements of Medicine of *John Brown*, M. D. tranflated from the Latin; with Comments and Illuftrations, by the Author. A New Edition, revifed, and corrected; with a Biographical Preface by *Thomas Beddoes*, M. D. and a Head of the Author. 2 vols. 8vo. *Johnfon*, London, 1795.

22. Medical Effays and Obfervations, with Difquifitions relating to the Nervous Syftem. By *James Johnftone*, M. D. Phyfician in Worcefter. And an Effay on Mineral Poifons. By *John Johnftone*, M. B. Phyfician in Birmingham; of Merton College, Oxford; Fel. of the Royal Med. Soc. Edin. Cor. Mem. of the Med. Soc. Lon. and late Phyf. to the Gen. Infir. Worc. 8vo. Evefham, 1795.

23. A fhort Account of the Origin, Symptoms, and moft approved Method of treating the putrid bilious Fever, vulgarly called the Black Vomit; which appeared in the City of the Havanna, with the utmoft Violence, in the Months of June, July, and part of Auguft, 1794; as practifed by Mr. *John Holliday*, an Englifh Surgeon, refident in that City. 8vo. Falmouth, 1795.

24. An

24. An Eſſay on the malignant peſtilential Fever, introduced into the Weſt-Indian Iſlands, from Boullam, on the Coaſt of Guinea, as it appeared in 1793 and 1794. By *C. Chiſholm*, M. D. and Surgeon to His Majeſty's Ordnance in Grenada. 8vo. *Dilly*, London, 1795.

25. A Treatiſe on the epidemic puerperal Fever of Aberdeen. By *Alexander Gordon*, M. D. Phyſician to the Diſpenſary. 8vo. *Robinſons*, London, 1795.

26. A Deſcription of the Jail Diſtemper, as it appeared amongſt the Spaniſh Priſoners, at Wincheſter, in the Year 1780; with an Account of the Means employed for curing that Fever, and for deſtroying the Contagion which gave riſe to it. By *James Carmichael Smyth*, M. D. F. R. S. Fellow of the Royal College of Phyſicians, and Phyſician Extraordinary to His Majeſty. 8vo. *Johnſon*, London, 1795.

27. An Account of the Experiment made at the Deſire of the Lords Commiſſioners of the Admiralty, on board the Union Hoſpital Ship, to determine the Effect of the Nitrous Acid in deſtroying Contagion, and the Safety with which it may be employed: in a Letter addreſſed to the Right Hon. Earl Spencer, By *James Carmi-*

*chael*

*chael Smyth*, M. D. F. R. S. &c. 8vo. *Johnson*, London, 1796.

28. Medical Reports of the Effects of Blood-letting, Sudorifics, and Bliftering, in the Cure of acute and chronic Rheumatifm. By *Thomas Fowler*, M. D. of York, Member of the Royal Medical Society of Edinburgh. 8vo. *Johnfon*, London, 1795.

29. Directions for Warm and Cold Sea Bathing; with Obfervations on their Application and Effects in different Difeafes. By *Thomas Reid*, M. D. F. A. S. 8vo. *Cadell* and *Davies*, London, 1795.

30. Hints refpecting the Chlorofis of Boarding Schools. By the Author of Hints refpecting the Diftreffes of the Poor. 8vo. *Dilly*, London, 1795.

31. An Inquiry into the Hiftory, Nature, Caufes, and different Modes of Treatment hitherto purfued, in the Cure of Scrophula and Cancer. By *William Nifbet*, M. D. Fellow of the Royal College of Surgeons, Edinburgh. 8vo. Edinburgh, 1795.

32. Practical Obfervations on the Natural Hiftory and Cure of the Venereal Difeafe. In Three Volumes. By *John Howard*, Surgeon. 8vo.

8vo. *Baldwin*, London, Vols. I. II. 1787. Vol. III. 1794.

33. A popular View of the Effects of the Venereal Disease upon the Constitution: collected from the best Writers. To which are prefixed, miscellaneous Observations, by a Physician. 8vo. Edinburgh, 1794.

34. Observations on morbid Poisons, Phagedæna, and Cancer: containing a comparative View of the Theories of Dr. Swediaur, John Hunter, Messrs Foot, Moore, and Bell, on the Laws of the Venereal Virus; and also some preliminary Remarks on the Language and Mode of Reasoning adopted by medical Writers. By *Joseph Adams*, of London, Surgeon. 8vo. *Johnson*, London, 1795.

35. Observations concerning the Prevention and Cure of the Venereal Disease; intended to guard the Ignorant and Unwary against the baneful Effects of that insidious Malady. With an Appendix, containing a List of the most approved Medicines now used in the Cure of this Disorder; also their Doses, Manner of Application, &c. By *William Buchan*, M. D. Fellow of the Royal College of Physicians, Edinburgh; and Author of the Domestic Medicine. 8vo. *Chapman*, London, 1796.

36. A Differtation on the Difeafes of Prifons and Poor Houfes; publifhed at the Requeft of the Medical Society of London, having obtained the Premium offered by the Society for the beft Effay on this Subject. To which is added a fingular Cafe of preternatural Fœtation, with Remarks on the Phenomena that occurred. Read before the Society, Oct. 20, 1794. By *John Mafon Good*, F.M.S. 12mo. *Dilly*, London, 1795.

37. The Hiftory of Medicine, fo far as it relates to the Profeffion of the Apothecary, from the earlieft Accounts to the prefent Period: the Origin of Druggifts, their gradual Encroachments on compound Pharmacy, and the Evils to which the Public are thence expofed; as alfo from the unfkilful Practice of ignorant Medicafters; and the Means which have lately been devifed to remedy thofe growing Abufes. Publifhed at the Requeft of the General Pharmaceutic Affociation of Great Britain. By *John Mafon Good*, Fellow of the Medical Society of London. 12mo. *Dilly*, London, 1795.

38. Murepfologia; or the Art of the Apothecary traced up to its original Source in Hiftory; and the Antiquity and Confequence of the Druggifts and Drug Merchants afferted and maintained tained against the Misrepresentations of the Author of a late History of Medicine. The Nature and Design of that Publication examined, and the true Foundation of the respectable Character of the Apothecary, of Great Britain, at the present Time, pointed out and illustrated. By *Joseph Bradney*, Esq. 8vo. *Rivingtons*, London, 1796.

39. Hints on the proposed medical Reform. By a Member of the Corporation of Surgeons. 8vo. *Johnson*, London, 1796.

40. A general View of the Establishment of Physic as a Science in England, by the Incorporation of the College of Physicians, London; together with an Inquiry into the Nature of that Incorporation; in which it is demonstrated, that the Exclusion of all Physicians, except the Graduates of Oxford and Cambridge, from the corporate Privileges of the College, is founded in Usurpation, being contrary to the Letter and Spirit of its Charter. By *Samuel Forris*, M. D., F. S. A. 8vo. *Johnson*, London, 1795.

41. Observations tending to show the Mismanagement of the medical Department of the Army; with a View to trace the Evils to their Source; and to point out to Government the Necessity of attending more to the Health of the Soldier in time of War. To which is annexed, a Reprefentation of the Syftem adopted in the Hanoverian Service. By *N. Sinnot*, M. D. 8vo. *Murray* and *Highly*, London, 1796.

42. A Letter to the Officers of the Army under Orders for, or that may hereafter be fent to the Weft Indies, on the Means of preventing that fatal Difeafe the Yellow Fever. By *Stewart Henderfon*, Surgeon of His Majefty's 40th Regiment of Foot, and many Years a Surgeon in the Royal Navy. 8vo. *Stockdale*, London, 1795.

43. An Inquiry into the Abufes of the Medical Department in the Militia of Great Britain, with fome neceffary Amendments propofed. Addreffed to the Prefident and Members of the Militia Club. By *H. Moifes*, Surgeon. 8vo. *Murray*, London, 1794.

44. Memoirs of the Medical Society of London; inftituted in the Year 1773, Vol. IV. 8vo. *Dilly*, London, 1795.

45. Medical Commentaries for the Year 1795; exhibiting a concife View of the lateft and moft important Difcoveries in Medicine and Medical Philofophy: collected and publifhed by *Andrew Duncan*, M. D. F. R. and A. S. S. Ed. Phyfician to his R. H. the Prince of Wales for Scotland, &c. Decade fecond. Vol. X. 8vo. Edinburgh, 1795.

46. Ob-

46. Obſervations on the Seats and Cauſes of Diſeaſes, illuſtrated by the Diſſections of the late Profeſſor *Morgagni*, of Padua. By *James Hamilton*, jun. M. D. Fellow of the Royal College of Phyſicians of Edinburgh. Vol. I. 8vo. Edinburgh, 1796.

47. On the Neceſſity of adopting ſome Meaſures to reduce the preſent Number of Dogs; with a ſhort Account of the Hydrophobia, and the moſt approved Remedies againſt it. By the Rev. *Edward Barry*, M. D. 8vo. Reading, 1796.

48. Eſſays and Obſervations, phyſiological and medical, on the Submerſion of Animals; and on the Reſin of the Acoroides reſinifera, or Yellow Reſin, from Botany Bay: to which are added, ſelect Hiſtories of Diſeaſes, with Remarks. By *Charles Kite*, 8vo. *Dilly*. London, 1795.

49. A new Inquiry into the Suſpenſion of vital Action, in Caſes of Drowning and Suffocation. Being an Attempt to concentrate into a more luminous Point of View the ſcattered Rays of Science, reſpecting that intereſting, though myſterious Subject. By *A. Fothergill*, M. D. F. R. S. &c. 8vo. *Rivingtons*, London, 1795.

50. An Eſſay on the Abuſe of ſpirituous Liquors; being an Attempt to exhibit, in its genuine Colours, its pernicious Effects upon the Property, Health, and Morals of the People; with Rules and Admonitions respecting the Prevention and Cure of this great national Evil. By *A. Fothergill*, M. D. F. R. S. &c. 8vo. Bath, 1796.

51. An Inquiry into the Nature and Properties of Opium; wherein its component Principles, Mode of Operation, and Use or Abuse in particular Diseases, are experimentally investigated; and the Opinions of former Authors on these Points impartially examined. By *Samuel Crumpe*, M. D. 8vo. *Robinsons*, London, 1793.

52. An experimental Essay on the Manner in which Opium acts on the living Animal Body. By *Alexander Philip Wilson*, M. D. Fellow of the Royal College of Physicians, and F. R. S. Edin. &c. 8vo. Edinburgh, 1795.

53. An Essay on the Rhus Toxicodendron, pubescent Poison Oak, or Sumach; with Cases shewing its Efficacy in the Cure of Paralysis and other Diseases of extreme Debility. By *John Alderson*, M. D. 8vo. Hull, 1794.

54. An Inquiry into the medical Efficacy of a new Species of Peruvian Bark, lately imported into this Country under the Name of Yellow Bark; including Practical Observations respecting ing the Choice of Bark in general. By *John Relph*, M. D. Phyſician to Guy's Hoſpital. 8vo. *Johnſon*, London, 1794.

55. A Letter on the Yellow Peruvian Bark, containing an hiſtorical Account of the firſt Introduction of that Medicine into France, and a circumſtantial Detail of its Efficacy in Diſeaſes; addreſſed to Dr. Relph, Phyſician to Guy's Hoſpital. By *Michael O'Ryan*, M. D. Late Profeſſor in the College of Lyons in France, and firſt Phyſician to the Grand Hotel Dieu of that City. 8vo. *Nunn*, London, 1794.

56. The Evidence of the Superior Efficacy of the Cinchona flava, or yellow Peruvian Bark: An Eſſay, in which the correſpondent Preparations of the three Peruvian Barks moſt generally known are compared; and in which the yellow is proved to excel the pale and the red, by that Evidence which is proper to Materia medica. By *Walter Vaughan*, M. D. Licentiate of the Royal College of Phyſicians, London; Phyſician at Rocheſter. To which is prefixed, a Letter to the Author, from Dr. *William Saunders*, F. R. S. and Senior Phyſician to Guy's Hoſpital. 8vo. *Johnſon*, London, 1795.

57. Obſervations on human and comparative

Parturition. By *R. Bland*, M. D. A. S. S. 8vo. *Johnson*, London, 1794.

58. Select Cases in Midwifery, extracted from the Records of the Edinburgh General Lying-in Hospital, with Remarks; by *James Hamilton*, jun. M. D. Assistant Physician to the Hospital, and Fellow of the Royal College of Physicians of Edinburgh. 8vo. Edin. 1795.

59. Domestic Midwife, or the best Means of preventing Danger in Child Birth. By *Margaret Stephen*, Teacher of Midwifery to Females. 12mo. *Fores*, London, 1795.

60. Rules* for recovering Persons recently drowned; in a Letter to the Rev. G. Rogers, A. M. 8vo. *Longman*, London, 1794.

61. The Anatomy of the Bones, Muscles, and Joints. By *John Bell*, Surgeon. 8vo. Edin. 1793.

62. Engravings, explaining the Anatomy of the Bones, Muscles, and Joints. By *John Bell*, Surgeon, Edinburgh. 4to. Edin. 1794.

63. An anatomical Description of the human gravid Uterus and its Contents. By the late *William Hunter*, M. D. Physician Extraordinary to the Queen, Professor of Anatomy in the Royal Academy, and Fellow of the Royal and

* By *R. Hamilton*, M. D.

Antiquarian

Antiquarian Societies, &c.. 4to. *Johnſon*, London, 1794.

64. The Works of the late Profeſſor *Camper*, on the Connexion between the Science of Anatomy, and the Arts of Drawing, Painting, Statuary, &c. In two Books: containing a Treatiſe on the natural Difference of Features in Perſons of different Countries and Periods of Life; and on Beauty, as exhibited in Ancient Sculpture; with a new Method of ſketching Heads, natural Features, and Portraits of Individuals with Accuracy, &c. Illuſtrated with Seventeen Plates, explanatory of the Profeſſor's leading Principles. Tranſlated from the Dutch, by *T. Cogan*, M. D. 4to. *Dilly*, London, 1794.

65. Experiments on the Nervous Syſtem, with Opium and Metalline Subſtances; made chiefly with the View of determining the Nature and Effects of Animal Electricity. By *Alexander Monro*, M. D. Profeſſor of Medicine, Anatomy, and Surgery, in the Univerſity of Edinburgh. 4to. Edinburgh, 1793.

66. Phyſiological Reſearches into the moſt important Parts of the Animal Oeconomy. Demonſtrating, 1. that the preſent Opinion concerning the Uſe of the Lymphatic Syſtem is erroneous, and that it does not terminate in the

Thoracic

Thoracic Duct. 2. The Difcovery of the great Importance and Ufe of the Lymph, of the Lymphatic Glands, and of the Lymphatic Syftem. 3. From the Difcovery of the Ufe of the Lymphatic Syftem, it is demonftrated how Poifons, &c. may be either received or prevented from entering into the Circulation by Abforption. 4. The Difcovery of the Ufe of the Brain and its Continuation, its Connexion with the Nerves, and with the Lymphatic Syftem. By *B. Humpage*. 8vo. *Murray*, London, 1794.

67. Zoonomia, or the Laws of Organic Life. By *Erafmus Darwin*, M. D. F. R. S. 2 Vols. 4to. *Johnfon*, London, 1794-6.

68. An Effay on the Caufes and Phenomena of Animal Life. By *John Herdman*, Member of the Medical Society, Edinburgh; and Surgeon in Leith. 8vo. Edinburgh, 1795.

69. A brief View of the anatomical Arguments for the Doctrine of Materialifm; occafioned by Dr. Ferriar's Argument againft it. By *William Tatterfall*, M. D. 8vo. *Johnfon*, London, 1794.

70. A Treatife on the Blood, or general Arrangement of many important Facts relative to the Vital Fluid. With fome curfory Obfervations on the Theory of Animal Heat. Interfperfed

ſperſed with pathological and phyſiological Remarks from the Inductions of modern Chemiſtry. By *Hugh Moiſes.* 8vo. *Evans,* London, 1794.

71. A Treatiſe on the Blood, Inflammation, and Gun-ſhot wounds. By the late *John Hunter.* To which is prefixed, a ſhort Account of the Author's Life, by his Brother-in-law, *Everard Home.* 4to. *Nicol,* London, 1794, with a Portrait of the Author and nine other Plates.

72. The Life of John Hunter. By *Jeſſe Foot,* Surgeon, 8vo. *Becket,* London, 1794.

73. Dialogues between a Pupil of the late John Hunter, and Jeſſe Foot; including Paſſages in Darwin's Zoonomia. 8vo. *Becket,* London, 1793.

74. Experiments on the inſenſible Perſpiration of the human Body, ſhewing its Affinity to Reſpiration; publiſhed originally in 1779, and now republiſhed with Additions and Corrections. By *William Cruikſhank.* 8vo. *Nicol,* London, 1795.

75. A phyſiological, theoretic, and practical Treatiſe on the important Utility and ſalutary Effects of the Science of muſcular Action; accompanied with plain and accurate Deſcriptions of the Muſcles of the human Body, in various various and different Points of View; diftinctly fhewing their refpective Motions as produced by Means of a peculiar and well-adapted Apparatus for reftoring the Power, Action, and Strength of the Limbs, whether difabled by Gout, Rheumatifm, Colds, paralytic Caufes, chronic Affections, Contractions or Sprains, or weakened by other Accidents, By *John Pugh.* 4to. *Dilly*, London, 1794, with 15 plates.

76. A practical Syftem of Surgery. By *James Latta*, Surgeon in Edinburgh. Illuftrated with Cafes on many of the Subjects, and with Copper Plates. 3 vols. 8vo. Edin. 1794-5.

77. A Dictionary of Surgery; or the young Surgeon's Pocket Affiftant. By *Benjamin Lara*, Member of the Corporation of Surgeons. 12mo. *Ridgeway*, London, 1796.

78. Difcourfes on the Nature and Cure of Wounds. By *John Bell*, Surgeon, 8vo. Edin. 1795.

79. A practical Effay on a certain Difeafe of the Bones, termed Necrofis, illuftrated with fix Plates. By *James Ruffell*, F. R. S. Edinburgh, Fellow of the Royal College of Surgeons, and one of the Surgeons of the Royal Infirmary, Edinburgh. 8vo. Edin. 1794.

80. The Hiftory of two Cafes of ulcerated

Cancer of the Mamma; one of which has been cured, the other much relieved, by a new Method of applying carbonic acid Air; illuſtrated by a copper Plate: with Obſervations. By *John Ewart*, M. D. one of the Phyſicians of the Bath City Infirmary and Diſpenſary. 8vo. *Dilly*, London, 1794.

81. A Treatiſe on the Hydrocele, on Sarcocele or Cancer, and other Diſeaſes of the Teſtes. By *Benjamin Bell*, F. R. S. Edin. 8vo. Edin. 1794.

82. Medical and Surgical Obſervations. By *Aug. Gottlieb Richter*, M. D. Profeſſor of Medicine in the Univerſity of Gottingen.--- Tranſlated from the German. 8vo. *Robinſons*, London, 1794.

83. Obſervations phyſiological and chirurgical on compound Fractures: containing an Anſwer to the following Queſtion, "What are the beſt Methods of treating compound Fractures, according to the Degree of Injury ſuſtained by the Limbs?" By *Walter Weldon*, Surgeon. 8vo. *Croſby*, London, 1794.

84. Hints reſpecting human Diſſections. 8vo. London, 1795.

85. Thoughts on the Practice of carrying off Bodies from Church Yards, &c. for Diſſection.

Dedicated

Dedicated to Sir John Frederick, Bart. 8vo. *Johnson*, London, 1795.

86. An Inquiry into the Causes which have most commonly prevented Success in the Operation of extracting the Cataract: with an Account of the Means by which they may either be avoided or rectified. To which are added, Observations on the Dissipation of the Cataract, and on the Cure of the Gutta serena. Also, additional Remarks on the Epiphora, or watery Eye. The Whole illustrated with a Variety of Cases. By *James Ware*, Surgeon. 8vo. *Dilly*, London, 1795.

87. Practical Observations on the Treatment of Strictures in the Urethra. By *Everard Home*, Esq. F. R. S. &c. 8vo. *Nicol*, London, 1795.

88. A new Method of operating for the femoral Hernia. Translated from the Spanish of Don *Antonio de Gimbernat*, Surgeon to the King of Spain. To which are added, with Notes by the Translator *, Queries respecting a safer Method of performing Inoculation, and the Treatment of certain Fevers. 8vo. *Johnson*, London, 1795. With two Plates.

89. Observations on the Causes of Distortions

* Dr. *Beddoes*.

of of the Legs of Children, and the Confequences of the pernicious Means generally ufed with the Intention of curing them: with Cafes to prove the Efficacy of a Method of Cure invented and practifed only by *T. Sheldrake*, Trufs-maker to the Weftminfter Hofpital and Mary-le-bone Infirmary. 8vo. *Egerton*, London, 1796.

90. A Short Addrefs to the Faculty, and others whom it may concern; recommending the Ufe of a new Poultice; with the View of faving wholly the Confumption of Bread and Oatmeal (at this Time in common Ufe for Poultices), being alfo a great faving in Expence, and attended with many other Advantages, not only to Public Hofpitals, and the Navy particularly, but to private Families. By *Thomas Payne*, Surgeon. Second Edition, with Alterations and Obfervations. 12mo. *Macleifh*, London, 1796.

91. Phamacopœia chirurgica; or Formulæ for the ufe of Surgeons: including, among a Variety of Remedies adopted in the private Practice of the moft eminent of the Profeffion, all the principal Formulæ of the different Hofpitals. 12mo. *Robinfons*, London, 1794.

92. The Works of *Charles Vial de Sainbel*, Profeffor of Veterinary Medicine. To which is prefixed, prefixed, a ſhort Account of his Life; including alſo the Origin of the Veterinary College of London. 4to. *Martin* and *Bain.* London, 1795.

93. Meteorological Obſervations and Eſſays. By *J. Dalton*, Profeſſor of Mathematics and Natural Philoſophy, at the New College, Mancheſter. 8vo. *Richardſon*, London, 1793.

94. On Electric Atmoſpheres. In which the Abſurdity of the Doctrine of poſitive and negative Electricity is inconteſtably proved: and the real Nature, Production, Mode of Exiſtence, and Properties of the Atmoſphere in an Electric State, are clearly demonſtrated and fully explained. To which is prefixed, a Letter, addreſſed to Mr. Read, of Knightſbridge, in Reply to his Remarks on the Author's former Tract on Electricity, &c. By *E. Peart*, M. D. 8vo. *Miller*, London, 1793.

95. A Meteorological Journal of the Year 1793; kept in London; by *William Bent*. To which are added, Obſervations on the Diſeaſes of each Month, in the City and Suburbs. 8vo. *Bent*, London, 1794.

96. A Comparative View of Meteorological Obſervations made in Ireland ſince the Year 1788; with ſome Hints towards forming Prognoſtics noſtics of the Weather. By *Richard Kirwan*, F. R. S. and M. R. I. A. 4to. Dublin, 1794.

97. Chemical Eſſays; being a Continuation of my Reflections on fixed Fire, with Obſervations and Strictures upon Drs. Prieſtley's, Fordyce's, Pearſon's, and Beddoes's late Papers in the Philoſophical Tranſactions; and an Anſwer to the Reviewers. By *Robert Harrington*, M. D. 8vo. *Faulder*, London, 1793.

98. A Summary of the pneumato-chemical Theory, with a Table of its Nomenclature, intended as a Supplement to the Analyſis of the new London Pharmacopœia. By *Robert White*, M. D. 8vo. *Cadell* and *Davies*, London, 1794

99. A Tranſlation of the Table of Chemical Nomenclature, propoſed by De Guyton, formerly De Morveau, Lavoiſier, Berthollet, and De Fourcroy; with Additions and Alterations. To which are prefixed, an Explanation of the Terms, and ſome Obſervations on the New Syſtem of Chemiſtry. By *George Pearſon*, M. D. 4to. *Johnſon*, London, 1794.

100. A Dictionary of Chemiſtry, exhibiting the preſent State of the Theory and Practice of that Science, its Application to Natural Philoſophy, the Proceſſes of Manufactures, Metallurgy, and numerous other Arts dependent on the Properties and Habitudes of Bodies in the

Mineral, Vegetable, and Animal Kingdom. With a confiderable Number of Tables, exprefsing the elective Attractions, fpecific Gravities, comparative Heats, component Parts, Combinations, and other Affections of the Objects of Chemical Refearch. By *William Nicholfon*. 2 vols. 4to. *Robinfons*, London, 1795.

101. The Philofophy of Chemiftry, or fundamental Truths of modern Chemical Science, arranged in a new Order. By *A. F. Fourcroy*, Tranflated from the French of the Second Edition. 8vo. *Johnfon*, London, 1795.

102. Effays, phyfiological and practical; founded on the modern Chemiftry of Lavoifier, Fourcroy, &c. with a View to the Improvement of the Practice of Phyfic. By *Francis Penrofe*, M. D. 8vo. *Deighton*, London, 1794.

103. An Effay on Combuftion, with a View to a new Art of Dying and Painting; wherein the phlogiftic and antiphlogiftic Hypothefes are proved erroneous. By Mrs. *Fulhame*. 8vo. *Johnfon*, London, 1794.

104. Letters from Dr. Withering, of Birmingham; Dr. Ewart, of Bath; Dr. Thornton, of London; and Dr. Biggs, of the Ifle of Santa Cruz; together with fome other Papers, fupplementary to two Publications * on Afthma,

* See Vol. V. page 195.

Con-

Confumption, Fever, and other Difeafes. By *Thomas Beddoes*, M. D. 8vo. Briftol, 1794.

105. Confiderations on the medicinal Ufe and on the Production of Factitious Airs. By *Thomas Beddoes*, M. D. and *James Watt*, Engineer. Parts I. II. III. 8vo. *Johnfon*, London, 1794-5.

106. Medical Extracts, being a concentrated View of fome of the late Difcoveries in Chemiftry, and the new Theory and Practice of Phyfic thereby introduced. By a Friend to Improvements. Vols. I. II. III. 8vo. London, 1794-5.

107. An Inquiry into the medicinal Qualities and Effects of the aerated alkaline Water; illuftrated by Experiments and Cafes. By *John Moncrieff*, Apothecary. 8vo. Edinburgh, 1794

108. A Short Account of the Nature and Properties of different Kinds of Airs, fo far as relates to their medicinal Ufe; intended as an Introduction to the pneumatic Method of treating Difeafes, with mifcellaneous Obfervations on certain Remedies ufed in Confumptions. By *Richard Pearfon*, M. D. Phyfician to the General Hofpital near Birmingham, and Member of the Royal College of Phyficians, London. 8vo. *Baldwin*, London, 1795.

109. Defcription of a pneumatic Apparatus, with Directions for procuring the factitious

Airs. By *James Watt*, Engineer. 8vo. *Baldwin*, London, 1795. With three plates.

110. Minutes * of the Society for Philosophical Experiments and Conversations. 8vo. *Cadell* and *Davies*, London, 1795.

111. The Antiphlogistic Doctrine of M. Lavoisier critically examined, and demonstratively confuted. In which its Absurdities are exposed, and clearly proved to arise from a Deficiency in its Principles; and that Defect is supplied; and an explanation given upon such Principles as Nature evidently employs, and Reason proves to be indispensably necessary. To which is added, an Appendix, consisting of Strictures on Dr. Priestley's Experiments on the Generation of Air from Water; and of Criticisms on the Remarks made by the Reviewers on the Author's former Writings. By *E. Peart*, M. D. 8vo. *Miller*, London, 1795.

112. Experiments and Observations relating to the Analysis of atmospherical Air; also farther Experiments relating to the Generation of Air from Water. Read before the American Philosophical Society, February 5th and 19th, 1796; and printed in their Transactions. To which are added, Considerations on the Doctrine of Phlogiston, and the Decomposition of Water, addressed to Messrs. Bertholler, &c. By *Joseph*

* By *Bryan Higgins*, M. D.

*Prieſtley*, L. L. D. F. R. S. &c. 8vo. Philadelphia printed; London re-printed, for *J. Johnſon*, 1796.

113. Chemico-Phyſiological Obſervations on Plants. By *M. Van Uſlar*. Tranſlated from the German, with Additions. By *J. G. Schmeiſſer*, F. R S. 8vo. Edin. 1795.

114. A Syſtem of Mineralogy, formed chiefly on the Plan of Cronſtedt. By *J. G. Schmeiſſer*, F. R. S. Vol. I. 8vo. *Dilly*, London, 1794.

115. Theſaurus Medicaminum. A new Collection of medical Preſcriptions, diſtributed into Twelve Claſſes, and accompanied with pharmaceutical and practical Remarks, exhibiting a View of the Materia Medica, and Practice of Phyſic, both at home and abroad. The ſecond Edition, with an Appendix and other Additions. By a Member of the London College of Phyſicians. 8vo. *Baldwin*, London, 1794.

116. Formulæ Medicamentorum ſelectæ. By the Author * of Maniacal Obſervations. 12mo. *Murray*, London, 1795.

117. A Pocket Conſpectus of the new London and Edinburgh Pharmacopœias: wherein the Virtues, Uſes, and Doſes, of the ſeveral Articles and Preparations contained in thoſe Works, are

* See vol. IV. p. 207.

conconcisely stated, their Pronunciation as to Quantity is distinctly marked, and a Variety of other Particulars respecting them given; calculated more especially for the Use of Junior Practitioners. By *Robert Graves*, M. D. of the Royal College of Physicians, London; Member of the Medical Societies of London and Edinburgh, &c. 12mo. *Murray* and *Highly*, London, 1796.

118. Transactions of the Linnean Society. Vol. II. 4to. *White*, London, 1794.

119. A Botanical Nomenclator, containing a systematical Arrangement of the Classes, Orders, Genera, and Species of Plants, as described in the new Edition of Linnaeus's Systema Naturæ, by Dr. Gmelin, of Gottingen. To which are added, alphabetical Indexes of the Latin and English Names of the Plants, together with the Names of the Countries of which they are Natives; also the Number of British Species. By *William Forsyth*, junior. 8vo. *Cadell*, London, 1794.

120. A Supplement to Medical Botany; or Part the Second: containing Plates, with Descriptions of most of the principal medicinal Plants not included in the Materia Medica of the Collegiate Pharmacopœias of London and Edinburgh; accompanied with a circumstantial Detail of their medicinal Effects, and of the Diseases in which they have been successfully employed.

employed. By *William Woodville*, M. D. F. R. S. Phyſician to the Small-Pox and Inoculation Hoſpitals. 4to. *Phillips*, London, 1794. With 64 plates.

121. The Natural Hiſtory of Aleppo. Containing a Deſcription of the City, and the principal natural Productions in its Neighbourhood; together with an Account of the Climate, Inhabitants, and Diſeaſes; particularly of the Plague. By *Alex. Ruſſell*, M. D. The Second Edition, reviſed, enlarged, and illuſtrated with Notes. By *Patrick Ruſſell*, M. D. and F. R. S. 2 Vols. 4to. *Robinſons*, London, 1794.

122. Plants of the Coaſt of Coromandel, ſelected from Drawings and Deſcriptions preſented to the Honourable Court of Directors of the Eaſt-India Company, by *William Roxburgh*, M. D. Publiſhed by their Order, under the Direction of Sir *Joſeph Banks*, Bart. P. R. S. Folio. Faſciculi I. II. III. *Nicol*, London, 1795-6.

123. A Naturaliſt's Calendar, with Obſervations in various Branches of Natural Hiſtory, extracted* from the Papers of the late Rev. *Gilbert White*, M. A. of Selborne, Hampſhire; ſenior Fellow of Oriel College, Oxford. 8vo. *Whites*, London, 1795.

124. Hortus Botanicus Gippovicenſis; or a ſyſtematical Enumeration of the Plants culti-

* By *John Aikin*, M. D.

vated in Dr. *Coyte's* Botanical Garden at Ipswich, in the County of Suffolk; also their generic Characters, English Names; the Natives of Britain particularized; the Exotics, where best preserved, and their Duration; with occasional Botanical Observations. To which is added, an Investigation of the Natural Productions of some Grass lands in High Suffolk. 8vo. Ipswich, 1796.

125. An Account of Indian Serpents, collected on the Coast of Coromandel; containing Descriptions and Drawings of each Species; together with Experiments and Remarks on their several Poisons. By *Patrick Russell*, M. D. F. R. S. Presented to the Hon. the Court of Directors of the East-India Company, and published, by their Order, under the Superintendence of the Author. Folio, with 46 coloured Plates. *Nicol*, London, 1796.

126. An Introduction to Botany: in a Series of familiar Letters, with Twelve Plates illustrative of the Work. By *Priscilla Wakefield*. 8vo. *Newbery*, London, 1796.

127. Memoirs of Science and the Arts; or an Abridgement of the Transactions published by the principal Learned and Oeconomical Societies established in Europe, Asia, and America. 4to. Vol. I. II. *Faulder*, London, 1793-4.

128. Dissertatio

128. Dissertatio inauguralis de Corpore Humano; Auctore *Carter Berkeley*, Virginiense. 8vo. Edinburgi, 1793.

129. Dissertatio inauguralis de Dysenteria; Auctore *Joanne Begg*, Jamaicense. 8vo. Edin. 1793.

130. Dissertatio inauguralis de Febre Scarlatina; Auctore *Malachia Blake*, Anglo. 8vo. Edin. 1793.

131. Dissertatio inauguralis de Hydrocephalo; Auctore *Joanne de Carro*, Genevense. 8vo. Edin. 1793.

132. Dissertatio inauguralis de Variola; Auctore *Josepho de Castro*, Lusitano. 8vo. Edin. 1793.

133. Dissertatio inauguralis de Amaurosi; Auctore *Joanne Crampton*, Hiberno. 8vo. Edin. 1793.

134. Dissertatio inauguralis de Tetano; Auctore *Richardo Crooks*, Jamaicense. 8vo. Edin. 1793.

135. Dissertatio inauguralis de Rheumatismo; Auctore *Hugone Duggan*, Hiberno. 8vo. Edin. 1793.

136. Dissertatio inauguralis de Oculo; Auctore *Alex. Edgar*, Scoto. 8vo. Edin. 1793.

137. Dissertatio inauguralis de Pertussi; Auctore *Roberto Halls*, Anglo. 8vo. Edin. 1793.

138. Dissertatio inauguralis de Blennorrhagia; Auctore *Gulielmo Hamilton*, Hiberno. 8vo. Edin. 1793.

139. Dissertatio inauguralis de Rubeola; Auctore *Francisco Harris*, Virginiense. 8vo. Edin. 1793.

140. Dissertatio inauguralis de Catarrho; Auctore *Andrea Huggan*, Scoto-Britanno. 8vo. Edin. 1793.

141. Dissertatio inauguralis de Phthisi; Auctore *Joanne Humberston*, Cambro-Britanno. 8vo. Edin. 1793.

142. Dissertatio inauguralis de Febre Intermittente; Auctore *Jacobo Maclennan*, Scoto-Britanno. 8vo. Edin. 1793.

143. Dissertatio inauguralis de Dysenteria; Auctore *Alexand. Menzies*, Scoto. 8vo. Edin. 1793.

144. Dissertatio inauguralis de Typho; Auctore *Carolo Minor*, Virginiense. 8vo. Edin. 1793.

145. Dissertatio inauguralis de Ischuria; Auctore *Francisco H. Northen*. Anglo. 8vo. Edin. 1793.

146. Dissertatio inauguralis de Colica Pictonum; Auctore *Samuele Pett*, Anglo. 8vo. Edin. 1793.

147. Dissertatio inauguralis de Chorea; Auctore *Joanne Butt Salt*, Anglo. 8vo. Edin. 1793.

148. Dissertatio inauguralis de Febre Puerperarum; Auctore *Gulielmo Simpson*, Anglo. 8vo. Edin. 1793.

149. Dissertatio inauguralis de Angina maligna; Auctore *Nicholao Sinnot*, Hiberno. 8vo. Edin. 1793.

150. Dissertatio inauguralis de Hysteria; Auctore *Gulielmo Belcher*, Anglo. 8vo. Edin. 1793.

151. Dissertatio inauguralis de Phthisi Pulmonali; Auctore *Thoma Cleghorn*, Britanno. 8vo. Edin. 1793.

152. Dissertatio inauguralis de Contagione humana. Auctore *Calebo Crowther*, Anglo. 8vo. Edin. 1793.

153. Dissertatio inauguralis de Phrenitide; Auctore *Jacobo Dubois*, Carolinense. 8vo. Edin. 1793.

154. Dissertatio inauguralis de Asthmate; Auctore *Saumarez Dubourdieu*, Hiberno. 8vo. Edin. 1793.

155. Dissertatio inauguralis de Variolis; Auctore *Pascale Paoli Drew*, Hiberno. 8vo. Edin. 1793.

156. Dissertatio inauguralis de Inflammatione; Auctore *Richardo Fowler*, Anglo. 8vo. Edin. 1793.

157. Dissertatio inauguralis de Rheumatismo; Auctore *Joanne Gamble*, Hiberno. 8vo. Edin. 1793.

158. Dissertatio inauguralis de Calorico; Auctore *Carolo Alex. Graham*, Scoto. 8vo. Edin. 1793.

159. Dissertatio inauguralis de Ascite; Auctore *Roberto Rogers Lowry*, Hiberno. 8vo. Edin. 1793.

160. Dissertatio inauguralis de Dyspepsia; Auctore *Joanne Mabarg*, Hiberno. 8vo. Edin. 1793.

161. Dissertatio inauguralis de Igne electreo; Auctore *Joanne Maculloch*, ex Insula Guernsey, 8vo. Edin. 1793.

162. Dissertatio inauguralis de Ictero; Auctore *Daniele Mills*, Hiberno. 8vo. Edin. 1793.

163. Dissertatio inauguralis de Cynanche maligna. Auctore *Jacobo Thompson Murray*, Hiberno. 8vo. Edin. 1793.

164. Dissertatio inauguralis de Ascite; Auctore *Gerardo Reumont*, Aquisgranense. 8vo. Edin. 1793.

165. Dissertatio inauguralis de Respiratione; Auctore *Gulielmo Russell*, Scoto. 8vo. Edin. 1793.

166. Dissertatio inauguralis de Febre intermittente; Auctore *Roberto Beverley Spratt*, Virginiense. 8vo. Edin. 1793.

167. Dissertatio inauguralis de Lumbricis; Auctore *Joanne Irvine Troupe*, Marylandinense. 8vo. Edin. 1793.

168. Delectus Opusculorum* ad omnem Rem medicam spectantium, quæ primum a celeberrimis

* Viz. 1. *Ant. Scarpa* de structura fenestræ rotundæ auris et de tympano secundario anatomicæ obs. 2. *J. B. Palletta* de nervis crotaphitico et buccinatorio. 3. *Leop. M. A. Caldani* de ureterum inequalitate et de fœtûs nutritione. Ej. Diss. de chordæ tympani officio; et de peculiari peritonæi structura. 4. *Laur. Nannoni* de similiarium partium humanum corpus constituentium regeneratione Diss. 5. *J. Bapt. Faleti* de abdita morbi causa per anatomen indagata in muliere infœcunda. 6. *Jo. A. Lapi* de acidula ad ripam Tyberis Epist. 7. *Paul Valcurenghi* de vera praxi medicis necessaria et ægrotis utili. 8. *Jo. Petr. Frank* oratio de populorum miseria, morborum genitrice. 9. *Petr. Orlandi* Diss. de variolarum refellenda inoculatione. 10. *Octav. Nerucci* hist. febris epidem. Senensis anni 1766.

 11. *Hier.*

rimis Italiæ Medicis edita, recudi curavit et præfatus est *Joannes Jacobus Römer*, Med. et Chir. Doctor. Vol. 1. 8vo. Zurich, 1791, c. tab. æn. VIII.

169. Pharmacopœia Amstelodamensis nova, 4to. Amstelodami, 1792.

170. *Joan. Frider. Weissenbornii*, Med. Prof. p. o. Rei obstetriciæ in Provincia Erfordensi et Nosocomii obstetricii Præfecti, &c. Observationes duæ de Partu Cæsareo; et Quæstiones de præcipuis hujus Operationis Momentis. 4to. Erfurt. 1792.

171. Dulcis Mercurii Laudes; Libellus medicus; Auctore *D. G. Frid. Hildebrandt*, M. Prof. 8vo. Erlangæ, 1793.

172. De vera Notione et Cura Morborum primarum Viarum Commentatio, cui alterum Præmium Ill. Academia Imp. Naturæ Curiosorum d. V. Jan. 1792, decrevit; Auctore D. *Gerardo Antonio Gramberg*, Rev. et Serenifs. Episc. Lubecens. Consiliario Cancellariæ, Aulæ Ducalis et Militiæ Medico. 8vo. Erlangæ, 1793.

11. *Hier. Mercurialis* nomothelasmus, sive ratio lactandi infantes. This last, which was first published at Padua in 1552, is here reprinted, it seems, on account of its scarcity. EDITOR.

173. Epis-

173. Epiſtolæ *Halleri* ad *Levelingium* ſcriptæ, quas edidit, præfatus eſt, notiſque illuſtravit *D. H. M. de Leveling* filius, S. R. I. Eques, Conſ. Elect. Act. Almæ Univerſitatis Ingolſtad. Anat. Phyſiol. Diætet. et Anthrop. Prof. p. o. &c. 8vo. Erlangæ, 1795.

174. *Jo. Frid. Blumenbachii*, Prof. Medic. ordin. M. Britann. R. Conſil. Aul. Societ. R. Scient. Gotting. aliarumque Membri, Decas altera Collectionis ſuæ Craniorum diverſarum Gentium illuſtrata. 4to. Goettingæ, 1793.

175. De Generis humani Varietate nativa. Editio tertia. Præmiſſa eſt Epiſtola ad Virum perilluſtrem *Joſephum Banks*, Baronetum, Regiæ Societatis Londin. Præſidem. Auctore *Jo. Frid. Blumenbach*, M. D. ejuſdem Societatis Sodali. 8vo. Goettingæ, 1795. c. tab. æneis II.

176. De Vi vitali Sanguini neganda Vita autem propria Solidis quibuſdam Corporis humani Partibus adſerenda Curæ iteratæ: quibus ſeptenis Medicinæ Candidatis digniſſimis, poſt habita Academicarum Diſputationum Specimina, Autumno A. pr. ſummos in Medicina Honores rite collatos eſſe indicit Ordinis medici tunc Temp. Decanus, *Jo. Frid. Blumenbach*,

M. D. et P. p. o. M. Britann. Regi a Consil. Aul. 4to. Goettingæ, 1795.

177. Hortus Gottingensis quem proponit simulque Orationem inchoandæ Professioni sacram indicit *Georg. Franc. Hoffman*, Med. Doct. Medic. Professor publ. ordinar. Horti R. Botan. Præfectus, Societatum Scientiarum Götting. Lugd. Phisiogr. Lund. Hist. Nat. Paris. aliarumque Membrum. Folio. Goettingæ, 1793, c. tab. æneis.

178. B. *Jo. Andreæ Murray*, Apparatus Medicaminum tam simplicium quam præparatorum et compositorum in Praxeos Adjumentum consideratus. Volumen primum. Editio altera auctior curante *Ludovico Christoph. Althof*, D. 8vo. Goettingæ, 1793.

179. Dissertatio inauguralis medica de Cortice Caribæo Cortici Peruviano substituendo; Auctore *Frederico Wilhelmo Ausmkolk*. 8vo. Goettingæ, 1793.

180. Dissertatio medica inauguralis de Hæmorrhoidibus; Auctore *Carol. Anton. Bitzio*. 8vo. Goettingæ, 1793.

181. Dissertatio inauguralis de Lue venerea complicata; Auctore *Isaaco Herz. Detmold*. 8vo. Goettingæ, 1793.

182. Dissertatio medica inauguralis de Salivationis vationis Usu in Morbis venereis; Auctore *Carolo Anton. Gloggner.* 8vo. Goettingæ, 1793.

183. Dissertatio inauguralis de Pulmonum Abscessu Ope chirurgica aperiendo; Auctore *Josepho Jacobo Gumprecht.* 8vo. Goettingæ, 1793.

184. Dissertatio inauguralis de Pertussi; Auctore *Joanne Harrison,* Anglo. 4to. Goettingæ, 1793.

185. Dissertatio medica inauguralis de Prolapsu ex Ano; Auctore *Gotl. Fried. Jordan.* 8vo. Goettingæ, 1793.

186. Dissertatio medica inauguralis de Struma; Auctore *Joh. Gerhard. Jordan.* 8vo. Goettingæ, 1793.

187. Dissertatio inauguralis de Icteri Pathologia; Auctore *Casp. Henrico Riemann.* 4to. Goettingæ, 1793.

188. Dissertatio inauguralis de Metastasibus Lactis; Auctore *Johanne Carolo Friderico Rust.* 4to. Goettingæ, 1793.

189. De nativo Vesicæ urinariæ inversæ Prolapsu Dissertatio inauguralis; Auctore *Theodoro Georgio Augusto Roose.* 4to. Goettingæ, 1793. c. tabulâ ænea.

190. Dissertatio inauguralis de Bronchotomia et et Œſophagotomia; Auctore *Edv. Fried. Tymm.* 8vo. Goettingæ, 1793.

191. Diſſertatio inauguralis de intempeſtivo Evacuantium Uſu in Febribus gaſtricis; Auctore *Fried. Carol. Voelckers.* 8vo. Goettingæ, 1793.

192. Diſſertatio inauguralis medica ſiſtens quædam de Methodo antigaſtrica ejuſque Noxa; Auctore *Chriſtiano Theophilo Steinecke,* Wiſmarienſe. 4to. Goettingæ, 1794.

193. Programma quo *Chr. Ehrener Weigel,* Ph. et Med. D. Chem. et Pharm. Prof. Reg. ord. Reg. Colleg. San. in Pom. Suec. et Reig. Direct. S. R. M. S. Archiater, &c. de Anthelminticis, et Euporiſto contra Tæniam quædam differit. 4to. Gryphiæ, 1795.

194. Collectio Diſſertationum medicarum minus cognitarum, habitarum in Academia Cæſar. Regia Leopoldina. 8vo. Inſpruck, 1793.

195. Specimen medicum Hiſtoriam ſiſtens Inſitionis Variolarum in Comitatibus Tecklaborgenſi atque Lingenſi exercitæ a *Leonardo Ludovico Finke,* M. D. Prof. p. o. 4to. Lingen, 1792.

196. *Fr. Boiſſier de Sauvages* Noſologia methodica ſiſtens Ægritudines Morbos Paſſiones

Ordine

Ordine artificiali ac naturali. Caſtigavit, emendavit, auxit, Icones etiam ad Naturam pictas adjecit *C. Fr. Daniel.* 8vo. Lipſiæ. Tom. II. 1790—1.

197. Hiſtoria pathologica ſingularis Turpitudinis Jo. G. Reinhardi, Viri 50 Annorum; Præfatus eſt *C. F. Ludewig*, Profeſſor Lipſienſis. Folio. Lipſiæ, 1793.

198. Theſaurus Materiæ medicæ et Artis pharmaceuticæ quem collegit atque edidit *Joann. Chriſt. Traugott Schlegel*, M. et Chir. D. Ser. Princip. de Schonburg Conſiliar. Aulic. et Archiater. Tom. Primus. c. tab. æn. 8vo. Lipſiæ, 1793.

199. Obſervationes pathologico-anatomicæ Auctarium ad Helminthologiam humani Corporis continentes; Auctore *Frederico Auguſto Treutler.* 4to. Lipſiæ, 1793. c. tab. æneis IV.

200. Quædam de Oſſibus foſſilibus Animalis cujuſdam, Hiſtoriam ejus et Cognitionem accuratiorem illuſtrantia; Auctore *Joanne Chriſtiano Roſenmueller*, Heſsberga-Franco. 4to. Lipſiæ, 1794.

201. De curandis Hominum Morbis Epitome, Prælectionibus academicis dicata; Auctore *Jo. Petr. Frank*, Therap. ſpecial. et Clinices in Ticinenſi Academia Profeſſor. Lib. I. de Febribus.

bribus. Lib. II. de Inflammationibus. Lib. III. de Exanthematibus. Lib. IV. de Impetiginibus. Lib. V. de Profluviis. 4to. Manheim, 1792—4.

202. *Caroli Friderici Clossii* Tractatus de Ductoribus Cultri lithotomi fulcatis. 8vo. Marburg, 1792.

203. Literatura universa Materiæ medicæ, alimentariæ, Toxicologiæ, Pharmaciæ, et Therapiæ generalis medicæ atque chiurgicæ potissimum academica. Scripsit *E. G. Baldinger*, Gulilelmo IX. Hassiae Landgr. Consil. intim. Archiater ord. Med. Marburg. Prof. primar. 8vo. Marburg, 1793.

204. *Jacobi Sacchi*, Phil. Med. et Chir. Doct. In Principia Theoriæ Brunonianæ Animadversiones. 8vo. Pavia, 1793.

205. Tabulæ nevrologicæ ad illustrandam Historiam anatomicam cardiacorum Nervorum, noni Nervorum Cerebri, glossopharyngæi et pharyngæi ex octavo Cerebri; Auctore *Antonio Scarpa*, Anat. et Chir. Clinices Professore, &c. c. tab. æn. XIV. Folio. Pavia, 1794.

206. Pathologia Therapiaque, quas in Usus suarum Prælectionum, præsertim ex Aphorismis magni Boerhavii, tum ex Operibus Gerardi van Swieten, Heisteri, &c. concinnavit *Matthæus*

*Collin*,

*Collin*, excelſi Regiminis Auſtriæ inferioris Conſiliarius, et in Univerſitate Vindobonenſi Profeſſor. 8vo. Viennæ, 1793.

207. Obſervationes medicæ varii Argumenti; præmittitur Methodus examinandi Aegros. Edidit *Joſephus Eyerel.* Sylloge I.—VI. 8vo. Viennæ, 1794.

208. *Joſephi Jacobi Plenck*, Hydrologia Corporis humani; ſive Doctrina chemico-phyſiologica de Humoribus in Corpore humano contentis. 8vo. Viennæ, 1794.

209. Oxalis. Monographia, Iconibus* illuſtrata; Autore *Nicolao Joſepho Jacquin.* 4to. Viennæ, 1794.

210. *Aſclepiadis Bithyni* Fragmenta. Digeſſit et curavit *Chriſtianus Gottlieb Grumpert.* 8vo. Weimar, 1794.

211. Specimen Semiologiæ medicinalis criticæ de Sopore interdum Periculi vacuo quin imo ſalutari; Auctore *J. Aug. G. Böttcher.* 8vo. Roſtoch, 1794.

212. Compendium pharmaceuticum caſtrenſibus Noſocomiis accommodatum. 4to. Pariſiis, 1792.

213. Precis ſur le Tetanos des Adultes; par par *N. Heurteloup*, Chirurgien consultant des Armées. 8vo. Paris, 1793.

214. Instruction relative à la Salubrité des Camps ; à la Santé et à la Conservation des Militaires : dont le Ministre de la Guerre a arreté l'Impression et l'Envoi aux Armées de la Republique, et dans les Garnisons ; avec Invitation à tous les Soldats Français, de suivre exactement ces Instructions vraiment salutaires : par les Membres du Conseil de Santé. 8vo. Paris, 1793.

215. Precautions relatives à la Dyssenterie dans les Armées ; par les Membres du Conseil de Santé. 8vo. Paris, 1793.

216. Instruction sur les Moyens d'entretenir la Salubrité et de purifier l'Air des Salles dans les Hôpitaux militaires de la Republique ; redigée par le Conseil de Santé du Departement de la Guerre ; en Execution du Decret de la Convention Nationale, du 14 Pluviose, de l'an 2$^{e}$. de la Republique une et indivisible ; approuvée le 7 Ventose, par le Conseil executif provisoire. 8vo. Paris, 1794.

217. Formulaire pharmaceutique à l'Usage des Hôpitaux militaires de la Republique Française. 8vo. Paris, 1794.

218. Essai sur la Topographie physique et medicale medicale de Paris ; ou Differtation fur les Subftances qui peuvent influer fur la Santé des Habitans de cette Cité, avec une Defcription de fes Hôpitaux; par le Citoyen *Audin Rouviere*, Officier de Santé, Membre de deux Sociétés libres d'Hiftoire Naturelle. 8vo. Paris, 1794.

219. Effai fur l'Utilité de la Chimie en Medecine ; par *Pierre Jofeph Delaville*, Medecin à Cherbourg. 8vo. Cherbourg, 1794.

220. Effai fur la Maladie qui affecte les Vaches laitieres des Fauxbourgs et Environs de Paris ; par le C. *Huzard*, Veterinaire. 8vo. Paris, 1794.

221. Inftruction fur les Moyens propres à prevenir l'Invafion de la Morve ; à en preferver les Chevaux, et a defінfecter les Ecuries où cette Maladie a regné ; par le C. *Huzard*. 8vo. Paris, 1794.

222. Rapport du Confeil de Santé, fur la Fouille des Terres des ci-devant Eglifes ; imprimé par Ordre du Comité de Salut publique de la Convention Nationale. 8vo. Paris, 1795.

223. Traité elementaire de la Medecine operatoire ; par *Pierre Laffus*. 2 vol. 8vo. Paris, 1795.

224. Traité complet d'Osteologie, suivant la Methode de *Desault* ; par *Hyacinthe Gavard*, son Eleve. Seconde Edition, augmentée du Traité des Ligamens. Tome 1. 8vo. Paris, 1795.

225. Instruction sur le Vertige abdominal, ou l'Indigestion vertigineuse des Chevaux ; des Caracteres auxquels on peut reconnoitre cette Maladie, les Moyens de la prevenir et de la combattre ; par *F. H. Gilbert*, Professeur Veterinaire. 8vo. Paris, 1795.

226. Instruction pour les Officiers de Santé des Corps armés, chargés de traiter, sous la Tente, les Militaires attaqués de Gales simples dans les Camps ; par les Inspecteurs generaux du Service de Santé des Armées. 8vo. Paris, 1796.

227. Instruction sommaire sur les Eaux minérales, à l'Usage des Troupes ; par les Inspecteurs généraux du Service de Santé des Armées : avec un Tableau des Hospices d'Eaux minerales, dont le Ministre de la Guerre a ordonné l'Etablissement à Portée des différentes Armées. 8vo. Paris, 1796.

228. Avis sur les Moyens de conserver, ou de rétablir la Santé des Troupes à l'Armée d'Italie par les Inspecteurs généraux du Service vice de Santé des Armées. 8vo. Paris. 1796.

229. Aufzüge aus dem Tagebuche eines ausübenden Arztes über verschiedene Gegenstände der Arzneywissenchaft. *i. e.* Extracts from the Journal of an experienced Physician relative to different Points of Medical Science. 8vo. Berlin, 1791.

230. Ausfurliche und genaue Beschreibung zweier höchst merkwürdiger und schwerer Geburtsfälle. *i. e.* A full and exact Description of two highly remarkable and difficult Cases of Midwifery; by *John Philip Hagen*, Professor of Midwifery at Berlin, &c. 8vo. Berlin, 1791.

231. Antisyphilitische Pharmakologie, oder Anleitung zur Kenntniss derjenigen rohen, zubereiteten und zusammem gesesften Arzneymittel, weiche bey der Heilung der Luftseuche pflegen angewendet zu werden. *i. e.* Antisyphilitic Pharmacology, or an Introduction to the Knowledge of the crude, prepared, and compound Remedies which are usually employed in the Cure of the Venereal Disease. By *Frederick Gott. Friese*, M. D. 8vo. Breslaw, 1791.

232. Ein paar Worte ueber die Faulfieber zu

Arzten und Nichtärzten gesprochen. *i. e.* Two Words on the Putrid Fever, addressed to Physicians, and to those who are not Physicians; by *C. H. Schobelt,* M. D. 8vo. Berlin, 1791.

233. Beytrage zur Litteratur der Blattern und deren Einimpfung, von Jahre 1768 bis 1790. *i. e.* Essays on the Literary History of the Small Pox and Inoculation, from the Year 1768 to 1790; by *Francis Olberg.* 8vo. Halle, 1791.

234. Beytrag zur Kenntnifs des Pemphigus. *i. e.* Help to the Knowledge of Pemphigus; by *John Ernest Wichmann,* M. D. Body Physician to his Majesty at Hanover. 4to. Erfurt, 1791.

235. Abhandlung von den anomalischen Gallenkrankheiten, die wahrender in der Graffschaft Tecklenburg, in den Jahren 1776—80, herrschenden Epidemie, beobachtet worden sind. *i. e.* An Essay on the Anomalous bilious Diseases which were observed in the Epidemic that prevailed in the County of Tecklenburg, in the Years 1776-80; by *L. L. Finke,* M. D. 8vo. Frankfort on the Mayne, 1791.

236. Abhandlung von der Verbindung der Luftseuche mit dem Scharbocke, und desselben Heilungsart. *i. e.* An Essay on the Combination tion of Syphilis with Scurvy, and the Method of treating it; by *Francis Schraud*, M. D. Physician at Segedin, in Hungary. 8vo. Vienna, 1791.

237. Aufsätze und Bemerkungen aus der praktischen Arzneywissenchaft und Geburtshülfe. *i. e.* Notes and Observations relative to the Practice of Physic and Midwifery; by *J. A. H. Zeller*, Physician at Malchin. 8vo. Leipsic, 1791.

238. Beschreibung des Weichselzopfs; nebst einer Anweisung, wie man sich in dieser Krankheit verhalten müsse, um davon zu genesen zum besten des Landvolks. *i. e.* A Description of the Plica Polonica; with Instructions relative to the best Method of treating this Disease among the Peasants; by *Jacob Frederick Hoffman*, M. D. translated from the Polish. 8vo. Konigsberg, 1792.

239. Chirurgisch-medicinische Abhandlungen verschieden inhalts-Polen betreffend. *i. e.* Chirurgo-medical Essays on different Subjects connected with Poland; by *F. L. de la Fontaine*, Surgeon to the King of Poland, &c. 8vo. Breslaw, 1792.

240. Vom Schaden der Brechmittel in der Lungensucht. *i. e.* On the ill Effects of Emetics in Phthisis; by *Anton. Fr. Metternich*, M. D.

Professor of Pathology at Mentz, &c. 8vo. Mentz, 1792.

241. Bemerkungen über die Durchbohrung des Processus Mastoideus in gewissen Fällen der Taubheit. *i. e.* Observations on the Perforation of the Mastoid Process in certain Cases of Deafness; by *Justus Arnemann*, M. D. Professor of Physic at Goettingen, &c. 8vo. Goettingen, 1792, with 3 copper-plates.

242. Grundriss der Zergliederungskunde des ungebohrnen Kindes in den verschiedenen Zeiten der Schwangerschaft. *i. e.* A Sketch of the Anatomy of the Fœtus in Utero at different periods of Pregnancy; by *Ferdinand George Danz*, M. D. &c. with Remarks by *S. T. Soemmering*, M. D. &c. 8vo. Frankfort, 1792.

243. Etwas über den Keichhusten als ein Beytrag zur Geschichte der Epidemieen des Jahrs 1780. *i. e.* Something relative to the Chin-Cough, as a Help to the History of the Epidemics of the year 1780; by *J. H. W. Klinge*, Physician at Osterode. 8vo. Goettingen, 1792.

244. Neue Versuche und Erfahrungen über einige Pflanzengifte. *i. e.* New Essays and Experiments on some Vegetable Poisons; by

*John Christ. Döltz:* publiſhed by *J. C. G. Ackermann,* M. D. &c. 8vo. Nurnberg, 1792.

245. Die Knochen des menſchlichen Körpers und ihre vorzüglichſten Bänder, in Abbildungen und kurzen Beſchreibungen. *i. e.* Delineations and ſhort Deſcriptions of the Bones of the Human Body, and their principal Ligaments; by *F. H. Loſchge,* M. D. and Prof. Folio. Erlangen, 1792.

246. Katechiſmus der Apothekerkunſt, oder die erſten Grundsätze der Pharmacie, für Anfänger. *i. e.* Catechiſm of the Art of the Apothecary, or firſt Principles of Pharmacy, for Beginners; by *Sigiſmund Frederick Hermbſtadt,* M. D. 8vo. Berlin, 1792.

247. Hippokrates Werke, aus dem Griechiſchen überſetzt und mit Erläuterungen. *i. e.* The Works of Hippocrates, tranſlated from the Greek, with Notes; by *John Fred. Charles Grimm,* M. D. Body Phyſician to the reigning Duke of Saxe Gotha. Vol. IV. 8vo. Altenburg, 1762.

248. Die Axe des weiblichen Beckens beſchrieben. *i. e.* The Axis of the female Pelvis deſcribed; by *John Chriſtopher Sommer,* M. D. Body Phyſician to the reigning Duke of Brunſ-

 wick.

wick. 8vo. Weiſſenfels, 1792; with a copper-plate.

249. Geſchichte der Botanik unſerer Zeiten. *i. e.* Hiſtory of the Botany of our Times; by *Frederick Caſimir Medicus.* 8vo. Manheim, 1793.

250. Briefe über Italien, vornemlich den gegenwartigen Zuſtand der Arzneykunde und die Naturgeſchichte betreffend. *i. e.* Letters on Italy, relative chiefly to the preſent State of Phyſic and Natural Hiſtory; addreſſed to Profeſſor *Sandifort* of Leyden, by *William X. Janſen*, Conſulting Phyſician to His Moſt S. H. the Elector Palatine, at Duſſeldorf. Tranſlated from the Dutch, with Additions, by the Author. 8vo. Duſſeldorf, 1793.

251. Bemerkungen über die zeitherige Gewohnheit hohe Beinkleider zu tragen, als eine bis jetzt nicht bemerkte Urſache öfterer Leiſtenbruche: nebſt der Beſchreibung einer neuen Art elaſtiſcher Bruchbänder, welche Leiſtenbruche, auch ſchon erwachſener Perſonen, radical heilen. *i. e.* Remarks on the preſent Cuſtom of wearing high Breeches, conſidered as one of the Cauſes, hitherto not obſerved, of the more frequent Occurrence of Inguinal Hernia: together with a Deſcription of a new Kind of of elastic Truss, by Means of which an Inguinal Hernia, even in Adults, may be radically cured. By *J. F. Weissenborn*, public Reader in Physic at Erfurt. 4to. Erfurt, 1793; with a copper-plate.

252. Chemische und Mineralogische Geschichte des Queckfilbers. *i. e.* Chemical and Mineralogical History of Quicksilver; by *George Fred. Hildebrandt*, M. D. Reader in Anatomy and Chemistry at Brunswick, &c. 4to. Brunswick, 1793.

253. Beweis, dass *Johann Mayow*, vor hundert Jahren den Grund zur antiphlogistischen Chemie und Physiologie gelegt hat. *i. e.* Proof that *John Mayow*, more than an hundred Years ago, laid the Foundation of the antiphlogistic Chemistry and Physiology*; by *John Andrew Scherer*, M. D. &c. 8vo. Vienna, 1793.

254. Beobachtung einer Ruhrepidemie im Meiningen im Monat Sept. und October 1791; nebst einem Anhang Witterungs Beobachtungen. *i. e.* Observations on an epidemic Dysentery at Meiningen in the Months of September and October, 1791; with an Appendix, containing Observations on the Weather: by

* See Volume II. page 203.

*George Henry Jawandts*, M. D. Phyſician at Meiningen. 8vo. Riga, 1794.

255. Von Baue des weiblichen Beckens. *i. e.* On the Structure of the female Pelvis; by *Charles Caſpar Creve*, M. D. 4to. Leipſic, 1794; with 9 copper-plates.

256. Beſchouwende en Werkende pharmaceutiſche, oeconomiſche, en Natuurkundige Chemie. *i. e.* The Theory and Practice of pharmaceutical, economical, and philoſophical Chemiſtry; by *P. J. Kaſtelein*, Apothecary at Amſterdam. 3 vols. 8vo. Amſterdam, 1786—93.

257. Efterretning om Trommeſygens Behandling hos Hornquaſget. *i. e.* Obſervations on the Treatment of the Tympany in horned Cattle; by *Erick Viborg*, Veterinary Profeſſor at Copenhagen, &c. 8vo. Copenhagen, 1792; with a copper-plate.

258. Forſog og Erfaringer om adſkitlige Gifters Virkning paa Dyr. *i. e.* Eſſay and Experiments on the Effects of different Poiſons on Animals; by *Erick Viborg*, Veterinary Profeſſor, &c. 4to. Copenhagen, 1792.

259. Oſſervazioni pratiche ſopra l'Amputazione degli Articoli, le invecchiarte Luſſazioni del Braccio, l'Idrocefalo, e il Panericcio, di *Giuſeppe*

*Giuseppe Flajani*, Dottore di Filosofia, e Medicina, Chirurgo di N. S. Papa *Pio* Sesto, Socio dell'Accademia delle Scienze di Siena, Lettore, Litotomo, e Chirurgo primario dell'Apostolico Archispedale di Santo Spirito in Sassia; Autore e Direttore del Museo Anatomico di detto Archispedale. 8vo. Rome, 1791.

260. Lettera di *Francesco Marabelli* concernente l'Esame dell'Acqua curata colla Paracentesi da un Idropico, della Clinica di Pavia nel Mese di Maggio dell'Anno 1791. 8vo. Pavia, 1791.

261. Riscontri fisico-botanici, ad Uso clinico, di *Andrea Comparetti*, P. P. P. nell'Universita di Padova. P. 1. 8vo. Padua, 1793.

262. Prodromo di Fisica vegetabile, di *Andrea Comparetti*, P. P. P. nell'Universita di Padova. 8vo. Padua, 1791.

263. Qualita ed Indicazioni diverse del Polso e della Orina nelle Malattie; Saggio di *Antonio Turra*, Medico-Filosofo. 8vo. Vicenza, 1792.

264. Ricerche intorno all'Acque minerali epatiche ed alla Analisi chimica di diverse Acque minerali dello Stato di Siena; di *Domenico Battini*, Pubblico Professore di Medicina pratica pratica nell'Universita di Siena. 8vo. Siena, 1793.

265. Ricerche fisiche del Dottore *Mattheo Zacchiroli* sulla Natura delle Acque, in cui se macerano le Canapi. 8vo. Fermo, 1793.

266. Della Melena, ossia del Morbo nero d'Ipocrate; Osservazioni di Dot. *Mattheo Zacchiroli*. 8vo. Imola, 1793.

267. Fisico Annale delle Acque e dei Bagni di Nocera; col Saggio di alcuni Articoli spettanti ad altre Acque minerali e ad altre Parti di Storia naturale del Territorio Noceriano. Anno primo, 1793. 8vo. Rome, 1793.

268. Farmacopea ad Uso dei Poveri *. 8vo. Milan, 1793.

269. Della Pazzia in genere, ed in ispezie. Trattato medico analitico con una Centuria di Osservazioni di *Vincenzo Chiarugi*, D. M. Professore di Medicina e Chirurgia nel R. Spedale di Bonifazio, Socio di diverse Accademie. Tomo primo. 8vo. Florence, 1793.

270. Lettera XI. Sull'Elettricita 'Animale; del Dottore *Eusebio Valli*, Corrispondente dell 'Accademia Reale delle Scienze di Torino. 8vo. Mantua, 1794.

* By Signor *Moscati*.

271. Osservazioni sulle Proprietà della China del Brasile, di *Andrea Comparetti*, P. P. P. 8vo. Padua, 1794.

272. Avviso al Popolo Toscano intorno all'Acidula della Selva; del Dott. *Cristofano Sarti*, Pubblico Professore del Collegio Ricci nell'Universita di Pisa. 8vo. Pisa, 1794.

273. Delle Acque minerali del Bergamasco; Trattato di *Giuseppe Pasta*, Protofisico di Bergamo, Socio di varie Accademie. 8vo. Bergamo, 1794.

274. Saggio di Osservazioni chirurgische di *Annibale Parea*, Medico Chirurgo ed Assessore della Regia Delegazione Medica di Varesa. 8vo. Varesa, 1794.

275. Dissertazioni di *Gaetano Strambia* sulla Pellagra. 8vo. Milan, 1794.

276. Fondamenti delle Scienza chimico-fisica applicati alla Formazione dei'Corpi ed ai'Fenomeni della Natura, esposti in due Dizionari, vecchio e nuovo de'fisico chimici. Con Tavole apposite indicanti l'Ordine d'un'utile Lettura. Opera di *Vincenzo Dandolo*, Veneto. 8vo. Venice, 1795.

INDEX.

### INDEX.

### A.

ABERNETHY, John, Account of Two Inftances of uncommon Formation of the Vifcera, — 100
Abfceffes, of the Lungs, Remarks on a Circumftance attending them, — — 133
Acid, Vegetable, and Sea Salt, a Mixture of, recommended in Dyfentery, — — 21
Adams, Jofeph, on Morbid Poifons — 321
Æther, Vitriolic, its Ufe in Typhus, — 11
———, Effects of the Vapor of, in Phthifis pulmonalis, — — 95
Alderfon, Dr. I. on the Rhus Toxicodendron, 326
Alexander, Difney, on the Cynanche Trachealis, 317
American Atmofphere, Obfervations on the Excefs of the Heat and Cold of, beyond that of Europe, — 225
Amputation, Work relative to, — 368
Amylaceous Matter, *fee* Starch.
Anatomy, Works relative to, 328, 329, 330, 333, 356, 364
Anguftura Bark, Obfervations relative to, — 41
Angina, Differtations on, — 347, 348
Animal Electricity, Works relative to, — 329, 370
Animation, fufpended, Works relative to, 325, 328
Arnold, Dr. Thomas, Cafe of Hydrophobia, 313
Arteries, their Importance in the Circulation of the Blood, confidered, — — — 187
Army, Works relative to the Difeafes of, 358, 360
Arnemann, J. on the Perforation of the Maftoid Procefs, 364
Afcites, Differtations on, — 348, 349
Afclepiades Bithynus, *fee* Grumpert.
Afthma, Differtation on, — — 347
Aufmkolk, F. W. de Cortice Caribæo, — 352

### B.

Bacon, Lord, his Account of the Converfion of Flefh into a fatty Subftance, — — — — 123

Baldinger, E. G. Literatura Materiæ medicæ, 356

Bark,

Bark, Peruvian, Works relative to, 326, 327, 371

——, Caribæan, Work relative to, —— 325

Barry, Dr. Edw. on the Hydrophobia, — 325

Battini, D. Ricerche intorno diverse Acque minerali, 369

Beddoes, Dr. Tho. Facts relative to the Origin of Intermittents, —— —— —— 26

——————, Translation of Dr. Brown's Elements of Medicine, —— —— 318

—————————— a Work on the femoral Hernia, —— —— —— 334

——————, Collection of Letters to, — 338

——————, on the med. Use of Factitious Airs, 339

Begg, Joannes, de Dysenteria, —— 345

Belcher, Gulielmus, de Hysteria, —— 347

Bell, Benj. prefers the Trepan to the Trephine, 195, 207

————, on Hydrocele and Sarcocele, — 333

——, John, Anatomy of the Bones, Muscles, and Joints, 328

————, Anatomical Engravings, —— ibid.

————, on the Cure of Wounds, —— 332

Bent, William, Meteorological Journal, —— 336

Berkeley, Carter, de Corpore humano, —— 345

Bile, secreted by an Artery, Instance of, —— 104

——, Dissertation on, — — — — 313

Bitzius, C. A. de Hæmorrhoidibus, —— 352

Black Vomit, Use of Cayenne Pepper in, — 11

——————, Work relative to it, —— 318

Bladder, Urinary, Dissertation on the Inversion of, 353

Blake, Malachias, de Febre Scarlatina, — 345

Bland, Dr. R. on human and comparative Parturition, 327

Blennorrhagia, Dissertation on, —— 346

Blood, Treatises on, —— —— 330, 331

Blood Vessels, unusual State of, in the human Body 101

Blumenbach, J. F. Craniorum Decas, —— 351

——————, de Generis humani Varietate, ibid.

——————, — Vi vitali Sanguini neganda, ibid.

Bones, of the human Body, Works relative to, 328, 365

——, fossil, of a certain Animal, Dissertation on, 355

Botany, Works relative to, 342, 343, 344, 352, 357, 366, 369

Böttcher, I. A. G. de Sopore, —— 357

Bradney, Joseph, Murepsologia, —— 322

Breeches, high, Remarks on the ill Effects of, 366

Bronchotomy,

Bronchotomy, Differtation on, ——— 353
Brown, Dr. John, Elements of Medicine, — 318
———, Animadverfions on his Theory, 356
———, Sir Thomas, his Account of a fatty Concretion found in a dead Body, ——— ——— 122
Buchan, Dr. W. on the Venereal Difeafe, — 321
Burns, Cafes of, cured by negative Electricity, 279
Burrowes, Dr. Geo. Cafe of an enlarged Spleen, 219

### C.

Cæfarean Operation, Work relative to, — 350
Calomel, its Ufe in Fevers, ——— 4, 5, 24
———, ——— the Yellow Fever, ——— 10
———, ——— Intermittents, ——— 14
———, ——— remitting Fevers, ——— 15
———, ——— Hepatitis, ——— 17
———, ——— Pleurify and Peripneumony, 19
———, ——— Dyfentery, ——— 20, 22
———, has long been employed in Fevers, in Jamaica, 24
Camper, Profeffor, on the Connexion between Anatomy and Painting, ——— ——— 329
Canal, Alimentary, uncommon Formation of, 106
Cancer, Works relative to, ——— ——— 320, 332
Carlifle, Anthony, Obfervations on Corns, — 29
Carro, Joannes de, de Hydrocephalo, ——— 345
Caffada, bitter, poifonous Quality of the Root of, 289
Caftration, performed by a Man on himfelf, Cafe of, 74
Caftro, Jos. de, de Variola, ——— 345
Cataract, Work relative to the Extraction of, 324
Catarrh, Differtation on, ——— ——— 346
Cayenne Pepper, its Efficacy in Typhus. — 11
———, — Ufe in counteracting the Effects of certain Poifons, ——— ——— ——— 290
Charcoal, Account of the external Ufe of, in Ulcers, 77
Chemiftry, Works relative to, 337, 338, 339, 340, 341, 368, 369, 370, 371
———, Dictionary of, ——— ——— 337
Chefelden, Mr. his Opinion of the Trephine and the Trepan, ——— ——— ——— 198, 201
Chiarugi, V. della Pazzia, ——— ——— 370
Children, an Effay on the Nurfing and Difeafes of, 314
Chin Cough, Works relative to, — 346, 353, 364

Chifholm,

Chisholm, Dr. C. on the Yellow Fever, — 319

Chlorosis, Work relative to, —— 320

Chorea, Dissertation on, —— —— 347

Cicuta, Effects of an æthereal Tincture of, in Phthisis, 98

Clark, Dr. James, on the Poison of bitter Cassada, 289

——, ———, ——- amylaceous Matter of different Vegetables, —— —— —— 300

Clarke, Dr. John, of an extraordinary Production of human Generation, —— —— —— 109

——-, ———, Remarks on the Effects of extraneous Substances introduced into the Body, — 87

Cleghorn, Dr. Geo. Remarks on Abscesses of the Lungs, 133

————, Thomas, de Phthisi pulmonali, — 347

Clossius, C. F. de Ductoribus Cultri lithotomi fulcatis, 356

Clutterbuck, Henry, on the Poison of Lead, 314

Cogan, Dr. T. his Translation of a Work by Professor Camper, —— —— —— 329

Cold Water, Use of, applied externally in Fevers, 2

Colica Pictonum, Dissertation on, —— 347

Collin, M. Pathologia et Therapeia, —— 356

Colon, uncommon Length of, in the human Body, 106

Comparetti, A. Riscontri fisico-botanici, — 369

————, —. Prodromo di Fisica vegetabile, *ibid.*

————, —. sulle Proprietà della China, — 371

Contagion, human, Works relative to, 319, 347

Corns, Observations on the Nature and Cure of, 29

Cows, Work on a Disease of, ——— 359

Coyte, Dr. Catalogue of his Botanic Garden, 343

Crampton, Joannes, de Amaurosi, —— 345

Creve, C. C. on the female Pelvis, —— 368

Crooke, Dr. Hezekiah, his Description of a Trephine, 206

Crooks, Richardus, de Tetano, ——— 345

Crowther, Caleb. de Contagione humana, — 347

Cruikshank, William, Expts. on the Nerves and their Reproduction, —— —— —— 136

————, ———, ——— insensible Perspiration, 331

Crumpe, Dr. S. on Opium, ——— 326

Cuticle, Observations on its Structure, —— 30

Cynanche Trachealis, Work relative to, —— 317

D.

Dalton, I. Meteorological Observations, — 336

Dandolo,

Dandolo, V. delle Scienza Chemico-fisica, — 371

Daniel, C. F. his Edition of the Nosol. Method. of Sauvages, — — — 354

Danz, F. G. on the Anatomy of the Fœtus in Utero, 364

Darwin, Dr. Erasmus, Zoonomia, — 330

Davidson, William, on the pulmonary System, 317

Delaville, P. J. sur l'Utilité de la Chimie en Medecine, 359

Detmold, I. H. de Lue venerea, — 352

Dexter, Dr. Aaron. Case of a locked Jaw, — 266

Dionis, his Directions in the Use of the Trepan, 196

———, Case in which he perforated the Scull twelve times, — — 199

Dissections, Work relative to, — 333

Dissertations, medical, Collections of, 350, 354, 355

Distortions, of the Legs of Children, Observations on, 335

Döltz, J. C. on Vegetable Poisons, — 364

Douglas, John, his Description of a Trepan, 193

Drew, Pascal Paoli, de Variolis, — 348

Dropsy, Dissertations on, — 348, 349

Drummond, Dr. his Use of Calomel in the Yellow Fever, 10

——— ——— the Pepper Medicine in the Black Vomit, — — 11

——— has long been in the habit of giving Calomel in Fevers, — — — 24

Dubois, Jacobus, de Phrenitide, — 347

Dubourdieu, Saumarez, de Asthmate — *ibid.*

Duggan, Hugo, de Rheumatismo, — 345

Duncan, Dr. Andrew, Medical Commentaries, 324

Dysentery, Observations relative to, — 19

———, its Fatality to Troops in the West Indies, *ibid.*

———, in the West Indies, generally accompanied with remitting Fever, — — 20

———, ———, how to be treated, *ibid.*

———, Works relative to, — 345, 358, 367

Dyspepsy, Dissertation on, — 348

E.

Ecchinus esculentus, Remarks on its Structure, 186

Edgar, Alexander, Dissertatio de Oculo, — 345

Electricity, negative, Effects of in Burns, — 279

———, Dissertation on, — — 348

Emetics, Work on the ill Effects of in Phthisis, 363

Emphy-

Emphysema, uncommon Case of, — 259

European Atmosphere, Observations on the Heat and Cold of, compared with that of America, — 225

Ewart, Dr. John, Cases of ulcerated Mamma, 333

Eye, Dissertation on, — 345

Eyerel, Josephus, Observationes medicæ, — 357

F.

Fallopius, his Precautions in the Use of the Chisel and Mallet in Fractures of the Scull, — 203

*Farine*, what so called in the West-India Islands, 304

Fatty Substance, Observations on the Conversion of Flesh into, — — — 121

Faust, B. C. Catechism of Health, — 316

Ferriar, Dr. John, Medical Histories, — 316

Ferris, Dr. S. on the Establishment of Physic as a Science in England, — — 323

Fever, Works relative to, 315, 318, 319, 345, 346, 347, 349, 354, 362.

Fienus, Tho. his Account of the Inconveniences of the Trepan, — — 195

Finke, L. L. de Insitione Variolarum, — 354

———, on anomalous bilious Diseases, 362

Fish, poisonous, Observations relative to, — 294

Flajani, G. on Amputation, &c. — 368

Fœtus, Remarks on the Powers and Functions of, 119

———, Work on the Anatomy of, — 364

Fontaine, F. L. de la, Medical Essays relative to Poland, 363

Fontana, Abbé, his Idea of the ultimate nervous Fibres, 157

Foot, Jesse, Life of John Hunter, — 331

Forbes, Murray, Treatise on Gravel and Gout, 313

Ford, Mr. his Account of a fatal Hæmorrhage from the Spleen in tapping, — — 222

Fordyce, Dr. George, on Simple Fever, — 315

Formulæ Medicamentorum selectæ, — 341

Forsyth, W. Botanical Nomenclator, — 342

Fothergill, Dr. A. on the Suspension of vital Action, 325

——— ——— Abuse of Spirituous Liquors, 325

Fourcroy, A. F. Philosophy of Chemistry, — 338

Fowler, Richardus, de Inflammatione, — 348

———, Dr. Tho. on acute and chronic Rheumatism, 320

Fractures, compound, Work relative to, — 333

Frank, I. P. de curandis Morbis, —— 355

Friese, F. G. Antisyphilitic Pharmacology, — 361

Fryer, Henry, Case of Pins extracted from the Breast of a Woman after remaining there Sixty Years, — 86

Fulhame, Mrs. Essay on Combustion, —— 338

G.

Gamble, Joannes, de Rheumatismo, —— 348

Garengeot, his Mode of using the Trepan, — 196

Gavard, H. Traité d'Osteologie, —— 360

Generation, human, Account of an extraordinary Production of, — — — — 109

Gibbes, G. S. on the Conversion of Animal Muscle into a Substance resembling Spermaceti, —— 121

Gimbernat, Don Anton. on the femoral Hernia, 334

Gloggner, C. A. de Salivationis Usu in Morbis venereis, 353

Golding, Widdows, of a remarkable Affection of the Testes, 62

——, ——, Case of a Man who castrated himself, 76

Gooch, Mr. Case in which he perforated the Scull Thirteen Times, — — — — — 199

Good, John Mason, on the Diseases of Prisons and Poor Houses, — — — — 322

——, —— ——, History of Medicine, — *ibid.*

Gordon, Dr. Alex. on puerperal Fever, — 319

Gout, Treatise on, —— —— 313

Graham, Car. Alex. de Calorico, —— 348

Gramberg, G. A. de Morbis primarum Viarum. 350

Gravel, Treatise on, —— —— *ibid.*

Graves, Dr. Robert, Instance of the fatal Effects of the Hemlock Dropwort, —— —— 308

——, —. ——, Conspectus of the London and Edinburgh Pharmacopœias, —— 341

Gregory, Dr. his successful Use of cold Water, externally, in Typhus, —— —— 2

Grimm, J. F. Works of Hippocrates, —— 365

Grumpert, C. G. Asclepiadis Bithyni Fragmenta, 357

Gumprecht, J. J. de Pulmonum Abscessu, — 353

H.

Hæmorrhoids, Dissertation on, —— 355

Hagen, J. P. two Cases of Midwifery, — 361

Haighton, Dr. John, on the Reproduction of Nerves, 155
Haller, his Letters to Leveling, —— 351
Halls, Robertus, de Pertuffi, — — — 346
Hamilton, Gulielmus, de Blennorrhagia, — *ibid.*
——, Dr. J. on the Seats and Caufes of Difeafes, 325
——, ——, Select Cafes in Midwifery, 328
——, —. R. on the Recovery of Perfons drowned, *ibid.*
Harrington, Dr. Robert, Chemical Effays, — 337
Harris, Francifcus, de Rubeola, —— 346
Harrifon, Joannes, de Pertuffi, —— 353
Heat, Differtation on the Principle of, —— 348
Heart, Inftance of an uncommon Tranfpofition of, 101
Heifter, his Precautions in the Ufe of the Trepan, 197
Helminthology, Work relative to, —— 355
Hemlock Dropwort, Inftance of its fatal Effects, 308
Henderfon, Stewart, on the Yellow Fever, — 324
Henthorn, James, Cafe in which he made three Perforations with the Trephine, — — 199
Hepatitis, Obfervations on, — — 17
——, acute, how to be treated, —— *ibid.*
——, chronic, fometimes miftaken for Dyfpepfia, *ibid.*
Hermbftadt, S. F. Pharmac. Catechifm, — 365
Hernia, inguinal, Work relative to, —— 366
Heurteloup, N. Precis fur le Tetanos, — 358
Higgins, Dr. B. Minutes of a Philofophical Society, 340
Hildebrandt, G. F. de Mercurio, — 350, 367
Hippocrates, German Tranflation of his Works, 365
Hoffmann, F. on the Plica Polonica, — 263
——, G. F. Hortus Goettingenfis, — 352
Holliday, John, on the Treatment of the Black Vomit, 318
Holyoke, Dr. E. A. on the Excefs of the Heat and Cold of the American Atmofphere beyond the European, 222
——, ——. Cafe of Emphyfema, — 259
Home, Everard, his Life of Mr. Hunter, — 331
——, ——, on Strictures of the Urethra, 334
Hooping Cough, Work relative to —— 317
Horfes, Works on the Difeafes of, —— 359, 360
Howard, John, on the Venereal Difeafe, — 320
Huggan, Andreas, de Catarrho, —— 346
Human Body, uncommon Formation of the Vifcera of, 101
Humberfton, Joannes, de Phthifi, —— 346

Humpage, B. Phyfiological Refearches, — 329
Hunter, John, Treatife on the Blood, — 331
———, ——, Life of, —— —— *ibid.*
————. Dr. W. Defcription of the gravid Uterus, 328
Huzard, C. fur une Maladie des Vaches, — 359
————, —. — la Morve, —— *ibid.*
Hydrocephalus, fingular Cafe of, —— 281
——————, Works relative to, — 314, 345
Hydrology, human, Work on, —— 357
Hydrophobia, Works relative to, — 313, 325
Hyfteria, Differtation on, —— —— 347

I.

Jacquin, N. J. Oxalis Defcriptio, —— 357
Janfen, W. X. Medical Letters on Italy, — 366
Jatropha Manihot, *fee* Caffada,
Jaundice, Differtations on, —— 348, 353
Jaw, locked, Cafe of, —— —— 266
Jawandts, G. H. on Dyfentery, —— 367
Inflammation, Differtation on, —— 348
Inoculation, of the Small Pox, Works relative to, 354, 362
Intermittents, Obfervations on the Treatment of, 12
——————, Facts relative to the Origin of, 26
——————, Works relative to, — 315, 346
Inteftines, uncommon Formation of, in the human Body, 106
Jones, John Gale, on the Hooping Cough, — 317
Johnftone, Dr. James, Medical Effays, — 318
————, —. John, on Mineral Poifons, — *ibid.*
Jordan, G. F. de Prolapfu ex Ano, —— 353
————, J. G. de Struma, —— —— *ibid.*
Ifchuria, Differtation on, — — 346
Italy, medical Works relative to, — 360, 366
Itch, Work relative to the Treatment of, in Camps, 360
Juvenal, mentions the freezing of the Tiber, as a common Event in his Time, —— —— 233

K.

Kaftelein, P. J. Theory and Practice of Chemiftry, 368

Key Inftrument, Defcription of a new one, — 90

Kidney, Cafe in which a Stone formed there was extracted through an Abfcefs in the Back, —— 285

King,

King, Samuel Croker, Defcription of an Inftrument for trepanning the Scull, —— —— 191
Kirwan, Richard, Meteorological Obfervations, 339
Kite, Charles, Effays Phyfiological and Medical, 325
Klinge, J. H. W. on the Chin-Cough, — 364

### L.

Lara, Benj. Dictionary of Surgery, —— 332
Laffus, P. Traité de la Medecine operatoire, 359
Latta, James, Syftem of Surgery, —— 332
Lavatio frigida, what fo called, —— 2
Lead, Poifon of, Work relative to, —— 314
Leveling, Letters to, from Haller, —— 351
Ligaments, Delineations and Defcriptions of, 365
Linnean Society, Tranfactions of, —— 342
Lithotomy, Work relative to the Inftruments of, 356
Liver, uncommon State of, in the human Body, 103
——, of Fifhes, Obfervations on the Circulation of the Blood in, —— —— —— 186
Livy, his Account of the fecond Punic War referred to, as a Proof of the Severity of the Winter Seafon, in Spain, at that Time, — — — — — 233
Locked Jaw, Cafe of, —— —— 267
Lofchge, F. H. on the Bones and Ligaments, 365
Lowe, P. his Remarks on the Operation of trepanning, 204
Lowry, R. R. de Afcite, —— 348
Ludewig, C. F. Hiftoria fingularis Turpitudinis, 355
Lumbrici, Differtation on, —— 349
Lungs, Remarks on an Appearance obferved on them, 133
——, Work relative to an Abfcefs of, —— 353
Lymphatic Syftem, Work relative to, — 314

### M.

Maclennan, Jacobus, de Febre Intermittente, 346

Maculloch, Joannes, de Igne electreo, — 348

Maharg, Joannes, de Dyfpepfia, —— *ibid.*

Marabelli, F. Efame dell'Acqua d' un Idropico, 369

Maftoid Procefs, Obfervations on the Perforation of, in Deafnefs, —— —— —— 364

Mayow, Dr. John, Work relative to, — 367

Meafles, Differtation on, —— 346

Medical Extracts, — — 339

——— Society, of London, Memoirs of, — 324

Memoirs of Science and the Arts, — 344

Menzies. Alex. de Dysenteria, — 346

Mercury, *See* Calomel.

———, Works relative to, — 350, 367

Mery, his Description of a Monster referred to, 175, 180, 182

——, his Opinion of the Circulation of the Blood between the Mother and Fœtus — *ibid.*

Meteorology, Work relative to, — 336

Metternich, A. F. on the ill Effects of Emetics in Phthisis, — — 363

Midwifery, Works relative to, 327, 328, 361, 363

Milk, Dissertation on the Metastases of, — 353

Mills, Daniel, de Ictero, — — 348

Mineralogy, System of, — — 341

Mineral Waters, Works relative to, 360, 369, 370, 371

Minor, Carolus, de Typho, — — 346

Moises, Hugh, on the Abuses of the Medical Department in the Militia, — — 324

———, Treatise on the Blood, — 330

Moncrief, John, on aerated alkaline Water, — 339

Monro, Dr. Alex. his Idea of the ultimate nervous Fibres, — — 157

——— Description of a human Male Monster, — — 170

———, referred to for an instance of the spleen being wounded in tapping, — 222

———, Experiments relative to Animal Electricity, — — 329

Monsters, Descriptions of two extraordinary ones, 109, 170

———, Remarks on, — — 113, 174

Moscati, Signor, Farmacopea ad Uso dei Poveri, 370

Moss, William, on the Nursing and Diseases of Children, — — 314

Murray, J. A. new Edition of his Apparatus Medicaminum, — — 352

———, J. T. de Cynanche maligna, — 348

Muscle, Animal, Observations on its conversion into a substance resembling Spermaceti, — 121

Muscular Action, Treatise on, — 331

N.

### N.

Necroſis, Eſſay on, — — 332

Negative Electricity, Effects of, in Burns, — 279

Nerves, Anatomical Tables of, — 356

——, Experiments on the Reproduction of, 136, 155

——, Inſtance of the Exiſtence of, where there was no Brain, — — 170, 187

Nervous Energy, not derived ſolely from the Brain and Cerebellum, — — 188

Nicholſon, William, Dictionary of Chemiſtry, 337

Niſbet, Dr. W. on Scrophula and Cancer, — 320

Nocera, Work on the Waters and Baths of, — 370

Nomenclator, Botanical, — 342

Northen, Franciſcus H. de Iſchuria, — 346

### O.

Oenanthe crocata. *See* Hemlock dropwort.
Oeſophagotomy, Diſſertation on, — 326
Olberg, F. on Small Pox and Inoculation, — 362
Opium, Works relative to it, — 326
Oxalis, Work on the Genus of, — 357

### P.

Paleſtine, Obſervations on the Climate of, 232, 234

Paré, Ambroſe, his Precautions in the Uſe of the Trepan, — — 197

Parea, Annib. Oſſervaz. chirurg. — 371

Paris, Work on the Medical Topography of, 358

Paſta, G. delle Acque min. del Bergamaſco, — 371

Pathology, Work relative to, — 356

Patterſon, Dr. William, on the Dropſy of the Brain, 314

—— —— Yellow Fever, 315

Payne, Thomas, Account of a new Poultice, 335

Pearſon, Dr. George, Tables of Chemical Nomenclature, — — 337

—— Richard, of the Effects of the vapour of Vitriolic Æther in Phthiſis, — 95

—— —— on different Kinds of Air, 339

Peart, Dr. E. on Electric Atmoſpheres, — 336

—— —— the Antiphlogiſtic Doctrine, 34[illegible]

Pellagra, Diſſertation on, — 371

Pelvis, female, Works relative to, — 365, 368

Pemphigus, Work relative to, ——— 362

Penrose, Dr. F. Essays physiological and practical, 338

Pepper medicine, how prepared, ——— 11

Peripneumony, of tropical Climates, Observations on, 18

Pett, Samuel, de Colica Pictonum, ——— 347

Pharmacopœia, Amstelodamensis, ——— 351

————, Castrensis, ——— 357

————, Chirurgica, ——— 335

————, Pauperum, ——— 370

Phrenitis, Dissertation on, ——— 347

Phthisis, Works relative to, — 346, 347, 363

Pins, remaining sixty Years under the Skin, instance of, 86

Placenta, Vessels of, supposed to have no Nerves, 117

Plenck, J. J. Hydrologia Corporis humani, 357

Pleurisy, of tropical Climates, Observations on, 18

Plica Polonica, Work relative to, ——— 363

Poisons, Essay on the Effects of different ones, on Animals, ——— ——— 368

————, Fish, Observations relative to, — 294

————, Vegetable, Remarks on, ——— 289, 267

————————, Work on, ——— 364

Poland, Medical Essays on Subjects relative to, 363

Pott, Mr. preferred the Trephine to the Trepan, 194, 198, 201

Prescriptions, medical, Collections of, — 341

Priestley, Dr. Joseph, on atmospherical Air, 340

Puerperal Fever, Dissertation on, ——— 347

Pugh, John, Treatise on muscular Action, 331

Pulse, Work relative to the State of, in Diseases, 369

R.

Rain, Observations on the annual quantity of, in different Countries, ——— — 242, 243

Reid, Dr. Tho. on Sea Bathing, ——— 320

Relph, Dr. John, on the Yellow Bark, — 326

Remitting Fevers, of tropical Climates, Obs. on, 14

Reproduction, of Nerves, Experiments on, 136, 155

Respiration, Dissertation on, ——— 349

Reumont, Gerardus, de Ascite, ——— *ibid.*

Rheumatism, Works relative to, — 320, 345, 348

Rhus Toxicodendron, Essay on, ——— 326

Richter, A. G. medical and surgical Observations, 333

Riemann, C. H. de Ictero, ——— 353

Roederer, Professor, his Description of a Monster, referred to, — 175, 177, 178, 179, 180, 182, 183

* Rome, the Climate of, shewn to be warmer now than formerly, ——— — 234

Romer, Joannes Jac. Delectus Opusculorum, 350

Roose, T. G. A. de Vesicæ urinariæ inversæ Prolapsu, 352

Rosenmueller, J. C. de Ossibus fossilibus, 355

Rouviere, A. sur la Topographie medicale de Paris, 358

Roxburgh, Dr. W. Plants of the Coast of Coromandel, 343

Rowley, Dr. W. rational practice of Physic, 316

Rust, J. C. F. de Metastasibus Lactis, ——— 353

Russell, Dr. Alex. History of Aleppo, — 343

———, Gulielmus, de Respiratione, ——— 349

———, James, on Necrosis, ——— 332

———, Dr. Patrick, his Edition of his Brother's History of Aleppo, ——— — 343

——————— Account of Indian Serpents, 344

Ryan, Dr. Michael O', on the Yellow Bark, 327

S.

Sacchi, J. de Theoria Brunoniana, ——— 356

Sainbel, Charles Vial de, Works of, — 335

Salt, J. B. de Chorea, ——— 347

Sarti, C. dell 'Acidula della Selva, ——— 371

Savigny, John, Description of a new Key Instrument, 90

Sauvages. F. B. Nosologia methodica, — 354

Scarlatina, Dissertation on, ——— 345

Scarpa, Anton. Tabulæ Nervorum, — 356

Scherer, J. A. Work relative to Dr. John Mayow, 367

Schlegel, J. C. T. Thesaurus Materiæ Medicæ, 355

Schmeisser, J. G. Chemic. phys. Observations on plants, 341

————————, System of Mineralogy, — *ibid.*

Schobelt, C. H. on Putrid Fever, — 361

Schraud, F. on the Combination of Syphilis with Scurvy, 363

Schutzercrants, Dr. H. Case in which a Stone formed in one of the Kidneys was extracted through an Abscess in the Back, — — 285

Scrophula, Work relative to, ——— 320

Sculteteus,

Scultetus, Cafe in which he perforated the Scull feven times, — — 199
Scurvy, Work relative to, — — 363
Sea Salt, and Vegetable Acid, a Mixture of, recommended in Dyfentery, — — 21
Sharpe, Mr. preferred the Trephine to the Trepan, 198, 201
Sheldrake, T. on the Diftortions of the Legs of Children, —— —— 335
Simmons, William, on the external Ufe of Charcoal in Ulcers, — —— 77
Simpfon, Gulielmus, de Febre Puerperarum, 347
Sinnot, Dr. N. on the medical Department of the Army, 323
————, de Angina maligna, —— 347
Sium nodiflorum, *See* Water Parfnep,
Small Pox, Works relative to, 313, 345, 348, 354, 362
Smith, Dr. of Savannah le Mar, his Ufe of Calomel in Fevers, —— —— 24
Smyth, Dr. J. C. on the Jail Diftemper, — 319
Soemmering, S. T. on the Anatomy of the Fœtus in Utero, 364
Solvents, Treatife on the Operation of, — 313
Sommer, J. C. on the Axis of the female Pelvis, 365
Spermaceti, converfion of Animal Mufcle into a Subftance refembling it, — — 121
Spigelia Anthelmia, its Efficacy in Worm Cafes, 297
Spinal Marrow, Experiments on, — 46
Spleen, enlarged, Inftances of, — 219, 222, 223
———, natural Size of, — — 221
———, Inftances of its being wounded in Tapping, 222
———, our Ignorance of its Ufe, mentioned, 223
Spratt, R. B. de Febre intermittente, — 349
Starch, Quantities of, yielded by different Vegetables, 300
Steinecke, C. T. de Methodo antigaftrica, 354
Stephen, Margaret, domeftic Midwife, — 328
Strambia, G. fulla Pellagra, —— 371
Struma, Differtation on, —— 353
Surgery, Works relative to, 332, 333, 334, 335
Syphilis, *See* Venereal Difeafe.

### T.

Tænia, Work relative to, — — 354
Tapioca, how prepared, — — 303
Tatterfall, Dr. W. on Materialifm, — 330
Tenghil, M. Cafe of Hydrocephalus, — 281
Teftes, remarkable Affection of, — 62
Tetanus, Cafe of, — — 266
——, Works relative to, — 345, 357
Thefaurus Medicaminum, — 341
Townfend, Rev. Jofeph, Phyfician's Vade Mecum, 315
——, Guide to Health, 316
Trafine, Derivation of the Word, — 216
Trepan, by whom preferred to the Trephine, 195, 201, 207
Trepanning the Scull, Defcription of an Inftrument for, 191, 213
——, *See* Bell, Chefelden, Crooke, Dionis, Douglas, Fallopius, Fienus, Garengeot, Gooch, Heifter, Henthorne, King, Lowe, Paré, Pott, Scultetus, Sharpe, Woodall.
Trephine, by whom preferred to the Trepan, 198
——, invented by Woodall, — 202
——, originally written *Trafine*, — 216
Treutler, F. A. de Helminthologia humani Corporis, 355
Troupe, J. Irvine, de Lumbricis, — 349
Turra, A. del Polfo e della Orina, — 369
Tymm, E. F. de Bronchotomia, — 353
Tympany, of horned Cattle, Obfervations on, 368
Typhus, Differtation on, — 346
——, Obfervations on the Treatment of, — 2

### U.

Valli, Eufebius, Sull'Elettricita Animale, 370
Vafcular Syftem, remarks on the Powers of, 118, 119
Vaughan, Dr. W. on the Yellow Bark, — 327
Veins, their Importance in the Circulation of the Blood, confidered, — — 187
Venereal Difeafe, Works relative to, 320, 321, 352, 361, 363

Viborg, Erick, on the Tympany of horned Cattle, 368

———————— Effects of different Poisons on Animals, —— — ibid.

Vinall, John, of the Effects of negative Electricity in Burns, —— — 279

Viscera, two Instances of uncommon Formation of, 100

Voelckers, F. C. de Febribus gastricis, — 354

Urine, Work on the Indications from it, in Diseases, 369

Uslar, M. Van, chem- phys. Observations on Plants, 341

Uterus, gravid, anatomical Description of, 328

W.

Wakefield, Priscilla, Introduction to Botany, 344

Wallis, Dr. George, Art of Preventing Diseases, 313

Ware, James, on the Cataract, ——— 334

Water Parsnep, Obs. relative to it, —— 312

Watt, James, Description of a pneumatic Apparatus, 339

Weldon, Walter, on compound Fractures, 333

Weigel, C. E. de Euporisto contra Tæniam, 354

Weissenborn, J. F. de Partu Cæsareo, — 350

———————— on the Custom of Wearing high Breeches, —— — 366

West Indies, Observations on the Diseases of, 1

White, Gilbert, Naturalist's Calendar, — 343

———, Dr. R. pneumato-chemical Theory, 337

Wichmann, J. E. on Pemphigus, — 362

Wilson, Dr. Alexander, on Opium, — 326

Winslow, his Description of a Monster referred to, 175, 176, 180, 181, 182, 185

Winterbottom, Dr. T. M. Observations on the Angustura Bark, —— — 41

Withers, Dr. Tho. on the Errors of medical Education, 314

Woodall, John, Inventor of the Trephine, — 202

Woodville, Dr. W. Supplement to medical Botany, 342

Worms, *See* Lumbrici, Tænia, Helminthology,

Wright, Dr. William, Obs. on acute Diseases, 1

Y.

Yellow Fever, Observations on, ——— 6

————, date of its Appearance in Grenada, 7

————, how treated in the West Indies, 7, 9

Yellow Fever, ſeldom Attacks Negroes or Creoles, 8

————, Venæſection ſeldom proper in it, 10

————, Uſe of Mercury in, — 10, 11,

————, Works relative to, 315, 319, 324

Z.

Zacchiroli, M. ſulla Natura delle Acque in cui ſe macerano le Canapi. —— — 370

———— della Melena, —— *ibid.*

Zeller, A. H. on the Practice of Phyſic and Midwifery, 363

Zoonomia, or the Laws of Organic Life, — 33[illegible]

END OF THE SEVENTH VOLUME.

